# Self-collection of samples for group B streptococcus testing during pregnancy: a systematic review and meta-analysis

**DOI:** 10.1186/s12916-023-03186-x

**Published:** 2023-12-18

**Authors:** Sarah A. Borg, Jenny Cao, Phi-Yen Nguyen, Samia Aziz, Joshua P. Vogel

**Affiliations:** Maternal, Child and Adolescent Health Program, Burnet Institute, 85 Commercial Road, Melbourne, VIC 3004 Australia

**Keywords:** Group B streptococcus, Group B streptococcal infection, GBS, Group B strep, Self-sampling, Self-care, Self-care intervention, Pregnancy, Antenatal, Diagnostic accuracy

## Abstract

**Background:**

Sample self-collection for reproductive tract infection diagnosis has been found to offer greater convenience, privacy, autonomy, and expanded access to testing in non-pregnant adults. This review aimed to determine whether sample self-collection is as accurate as provider-collection for detection of group B streptococcus colonisation in pregnancy and whether a strategy of self-collection compared to provider-collection might improve maternal and neonatal health outcomes.

**Methods:**

We searched CINAHL Plus, Medline, EMBASE, Maternity and Infant Care Database, Cochrane Central Register of Controlled Trials, and Cochrane Database of Systematic Reviews in June 2022. Eligible studies compared self-collected and provider-collected samples taken from the same participants or participants randomised to either self-collection or provider-collection for reproductive tract infection testing using the same test and testing method in pregnant individuals. We included trials and observational studies. Reviewers assessed risk of bias using the QUADAS-2 checklist and independently extracted data. Sensitivity and specificity for group B streptococcus colonisation of self-collected compared to provider-collected samples were pooled using a bivariate, random-effects, meta-analytic model. This review was registered with PROSPERO (CRD42023396573).

**Results:**

The search identified 5909 references, of which eleven diagnostic accuracy group B streptococcus studies were included (*n* = 3269 participants). No studies assessed the effects of self-collection in pregnancy on health outcomes. All studies had high or unclear risk of bias. Pooled sensitivities of self-collected samples for group B streptococcus detection were 82% (95% CI: 66–91%; *I*^2^ = 68.85%) in four trials (*n* = 1226) and 91% (95% CI: 83–96%; *I*^2^ = 37.38%) in seven non-randomised studies (*n* = 2043). Pooled specificities were 99% (95% CI: 98–99%; *I*^2^ = 12.08%) and 97% (95% CI: 94–99%; *I*^2^ = 72.50%), respectively.

**Conclusions:**

Self-collected samples for group B streptococcus detection in pregnancy had high specificity compared to provider-collection, but lower sensitivity, particularly for included trials. Studies investigating the effect of self-collection on health outcomes, and further higher quality trials comparing accuracy of self-collection to provider-collection, are required.

**Supplementary Information:**

The online version contains supplementary material available at 10.1186/s12916-023-03186-x.

## Background

Group B streptococcus (GBS) is a commensal bacterium that is generally found in the gastrointestinal and genitourinary tracts of pregnant women [1] and can be passed to their baby via maternal rectovaginal colonisation during labour, causing neonatal early-onset GBS disease (EOGBS) [1, 2]. Maternal rectovaginal GBS colonisation varies between populations but is estimated to occur transiently in approximately 18% of pregnant women worldwide [3, 4]. GBS is a leading cause of adverse maternal and neonatal outcomes, including maternal and neonatal sepsis, stillbirth, and infant death [1].

Intrapartum antibiotic prophylaxis (IAP) can prevent EOGBS [5]. The World Health Organization (WHO) recommends IAP administration to women with GBS colonisation, within the context of local policy and guidance on GBS screening [4]. Some countries like the Netherlands and the United Kingdom recommend risk-based protocols, giving IAP only in the presence of peripartum clinical risk factors. Other countries, like the United States (US), recommend both risk-based and universal culture-based screening for GBS colonisation, so that IAP can be given in the case of known colonisation [5]. Culture-based testing remains the standard for antepartum screening [2]. There is currently no international consensus on whether to recommend risk-based or universal culture-based screening for GBS [5]. The need for laboratory processing limits testing capabilities in low-resource settings [6]. A 2020 systematic review and meta-analysis found that screening-based protocols were associated with a reduced risk of EOGBS compared to risk-based protocols, without an associated higher antibiotic administration rate [5]. If pregnant women do screen for GBS colonisation, it is important that the sampling approach has reasonable test accuracy, as false positives can contribute to overtreatment and resultant risk of antibiotic resistance, as well as having adverse effects on neonatal microbiome development [5]. Conversely, false negatives present a missed treatment opportunity to reduce the risk of maternal and infant morbidity and mortality [5].

GBS testing is most sensitive when performed near or at term [7]. GBS detection rates are higher when a combined vaginal-rectal swab is taken compared to a single vaginal or rectal swab only [8]. Testing is traditionally done with GBS culture, either by or direct plating and/or incubating the specimen in enriched culture medium. Enriched culture has a higher sensitivity than direct plating alone [9]. Nucleic acid amplification test (NAAT) methodology for GBS testing is available; however, it has limitations, such as the inability to perform susceptibility testing. NAAT has not yet been universally adopted [2].

WHO defines self-care as, “the ability of individuals, families and communities to promote health, prevent disease, maintain health and cope with illness and disability with or without the support of a health worker… Self-care interventions are tools that support self-care.” Self-care interventions include self-collection of samples [10]; this involves an individual taking their own specimen, which is sent to a laboratory for processing [11]. Systematic reviews comparing self-collected and healthcare provider-collected samples in the general population have found comparable accuracy for reproductive tract infection (RTI) testing, including sexually transmitted infections (STIs) [12] and human papillomavirus (HPV) [13]. A 2019 systematic review found that self-collection of samples for STI diagnosis in the general population offers convenience, confidentiality, expanded access, and increased patient autonomy and empowerment [11]. Self-sampling for STIs is acceptable to patients [14], and programmes offering self-collection have been found to increase uptake of STI testing [11, 15, 16] and case finding [11], without significant adverse outcomes [15]. This may not necessarily translate into increased uptake of screening for GBS during pregnancy, as barriers specific to STI screening, like stigma, may not be as significant an issue for GBS sampling, which is a routine part of antenatal care in some countries. However, screening enablers such as increased acceptability of and reduced embarrassment associated with self-sampling compared to provider-collection may be transferrable.

The US Centres for Disease Control and Prevention guidelines advise that, when paired with clear patient instructions, self-collected vaginal-rectal specimens in pregnancy have similar GBS culture yield rates to provider-collected specimens [2]. However, the supporting evidence for this advice are studies that have not been formally synthesised [17–20]. No previous systematic review has assessed the accuracy of self-collected samples for RTIs in pregnancy, including GBS, and whether self-collection of samples for RTIs in pregnancy can improve maternal and perinatal health outcomes.

Ensuring there is high quality evidence that sample self-collection for GBS screening is as accurate as provider-sampling presents an opportunity for a strengthened evidence base to support self-care interventions during pregnancy. Expansion of self-care in this context could improve patient choice, convenience, and autonomy, as well as expand antenatal care coverage, ultimately improving health outcomes [21]. This review aimed to determine (1) whether self-collected samples are as sensitive and specific as provider-collected samples for detection of GBS colonisation in pregnant individuals and (2) whether a self-collection strategy for detection of GBS in pregnancy compared to provider-collection can improve maternal and perinatal health outcomes.

## Methods

### Search strategy and assessment of eligibility

This systematic review and meta-analysis is part of a larger systematic review (PROSPERO CRD42023396573) which aims to determine the diagnostic accuracy and health effects of sample self-collection for RTI testing in pregnant individuals, compared to provider-collection. In this paper, we report on studies assessing GBS colonisation (results for other RTIs will be reported separately). We report these findings according to PRISMA-DTA guidelines (see Additional file 1 for PRISMA Checklist) [22].

Eligible studies included those comparing self-collected to provider-collected samples for GBS testing of pregnant individuals. We included studies whose population were entirely or partially comprised of pregnant individuals, provided that disaggregated data for pregnant participants could be obtained (either from the published article, or by contacting study investigators). Studies were eligible if self-collected and provider-collected samples were taken from the same participant or participants were randomised to either self-collection or provider-collection. To be eligible, studies needed to have all participant samples collected from the same anatomical site, with the same type of sampling device, following the same sample transport process, with the same sample processing and test performed on both samples, using the same test cut off. Eligible studies were those reporting positive and negative test results for both self-collected and provider-collected samples. Randomised or quasi-randomised controlled trials, controlled before-after studies, interrupted-time-series studies, historically controlled studies, cohort studies, cross-sectional studies and case–control studies were eligible. We excluded case reports, case series, conference abstracts, poster presentations, editorials, correspondence, and qualitative studies. For protocols of ongoing trials dated 2019 or later, we contacted authors to see if trial data were available.

The following databases were searched on 18–21 June 2022: CINAHL Plus via EBSCOhost (from 1937), Medline and EMBASE (from 1946), Maternity and Infant Care Database (from 1971), Cochrane Central Register of Controlled Trials (from 1998), and the Cochrane Database of Systematic Reviews (from 1996) via Ovid. The search strategy combined keywords and subject headings on self-care (including self-sampling and self-collection) AND pregnancy AND reproductive tract infections (see Additional file 2 for full search strategy). No date or language restrictions were applied. We conducted a manual search of the reference lists of systematic reviews on similar topics for non-pregnant participants, as well as those of included studies in this review.

Two reviewers independently screened all titles/abstracts and potentially eligible full texts for inclusion using Covidence, according to the eligibility criteria. Any disagreements were resolved through discussion or consulting a third reviewer. When warranted, Google Translate was used for studies not in English. For eligible randomised trials, two independent reviewers assessed trial integrity using an adapted research integrity assessment (RIA) checklist tool, which consists of six domains to assess trial research integrity [23, 24]. We reported findings from the RIA tool for each study by domain and contacted study authors for further information regarding these concerns.

### Data extraction, quality assessment, and analysis

Two reviewers independently extracted data and performed risk of bias assessment using an Excel-based form. Disagreements between individual judgements were resolved by a third reviewer. When multiple articles reported on the same study, data was combined into a single data extraction.

We extracted data on study characteristics—study location, sample size, eligible participants, sample collection process, including location and timing, anatomical site, specimen type, and sampling device, sample transport, stage medium, type of test, test threshold, and the number of GBS colonisation true positives (TP), false positives (FP), true negatives (TN), and false negatives (FN).

Our review outcomes (see Additional file 3) were diagnostic accuracy, and maternal, perinatal and neonatal outcomes. Additional secondary outcomes were outcome of sampling order, uptake of self-collection, case finding, linkage of positive test to clinical assessment or treatment, feasibility, and patient acceptability and preference. Missing or unclear information was noted as such. Where review outcome data were missing, we contacted study investigators to see if additional data were available. Risk of bias in the included studies and concerns regarding applicability to the review question were assessed independently by two reviewers using the QUADAS-2 checklist [25]. Any disagreements were resolved by a third reviewer.

Meta-analysis was conducted according to the Cochrane Handbook for Systematic Reviews of Diagnostic Test Accuracy, Version 2.0 [26]. Trials and non-trials were analysed separately. Revman 5.4 was used to generate forest plots for sensitivity and specificity. Other analyses were performed using Stata SE version 17 (STATA Corp., Texas, USA). Using the Stata command *metandi*, we calculated the sensitivity and specificity for each study and pooled these estimates using a bivariate, random-effects, meta-analytic model. This model is required to describe variability in accuracy between studies, due to expected heterogeneity [26]. A Hierarchical Summary Receiver Operating Characteristic (HSROC) plot was constructed using *metandi*, which provides a global summary of both sensitivity and specificity estimates and accounts for between-study variability [27]. The HSROC plot presented sensitivity and specificity estimates from individual studies, the pooled estimates, 95% confidence interval (CI), and 95% prediction interval, i.e. the confidence region for a forecast of true sensitivity and specificity in a future study [27].

Since no covariates were included in the meta-analysis, the bivariate and HSROC models were mathematically equivalent [28] and thus presented together. Stata’s *metadta* command was used to calculate the bivariate *I*^2^ value, which measures heterogeneity whilst accounting for the correlation between logit sensitivity and logit specificity [29]. Meta-regression was not done because there were less than ten studies in each meta-analysis [26]. There were insufficient or zero studies to undertake subgroup analyses by sample anatomical site, gestational age (GA), culture technique, or NAAT thresholds. Forest plots were instead examined visually for any trends in pre-specified subgroups of interest.

Sensitivity analyses were conducted to investigate the effect of non-randomised studies on the summary estimates which (1) included participants at < 35 weeks’ gestation (*n* = 3) and (2) did not incubate all culture samples in enriched culture media (*n* = 1). There were too few studies for further sensitivity analyses.

## Results

### Screening results

**Fig. 1:**
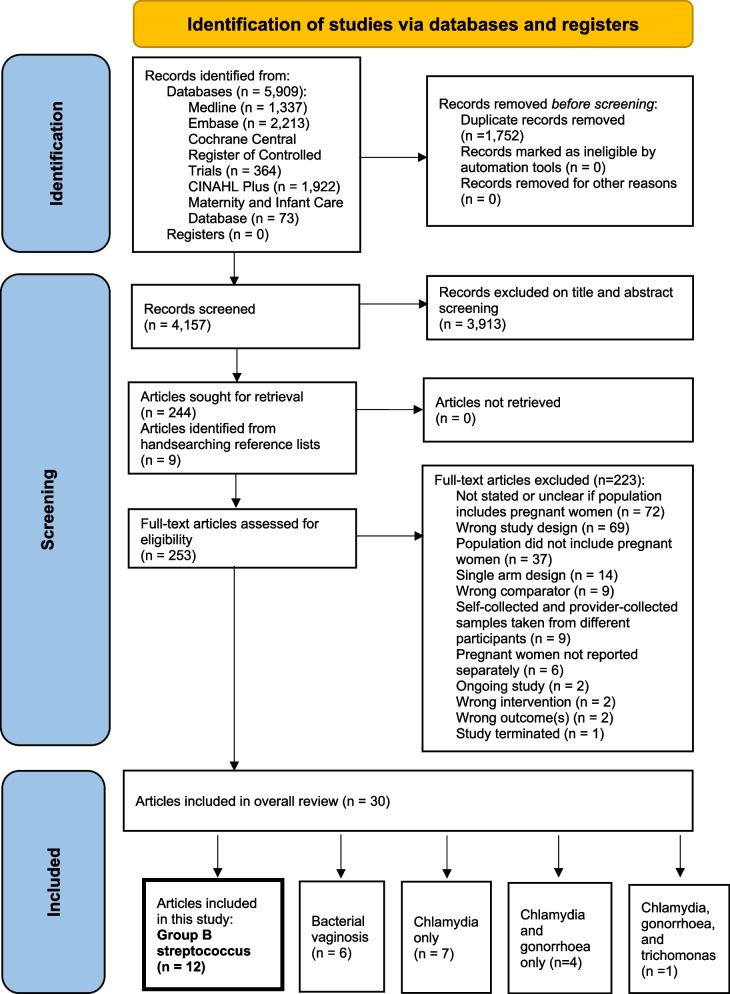
PRISMA flowchart [30] of identification and selection of studies

The search identified 5909 citations (Fig. 1). A total of 3913 citations were excluded following title/abstract screening. Nine further articles were retrieved from manual searching of reference lists. On full text review, 223 citations were excluded, with reasons outlined in Fig. 1 and Additional file 4, leaving 30 articles reporting on 5 RTIs.

### Characteristics of included studies

Eleven studies (3269 participants) reported in 12 articles [17, 19, 20, 31–39] related to GBS in pregnancy were included in the meta-analysis. Two articles [20, 38] published the results from the same study and were thus extracted collectively. Table 1 displays key study characteristics. Four were randomised cross-over trials [19, 31, 36, 39], and eight were non-randomised studies [17, 20, 32, 34, 35, 37, 38], including one non-randomised cross-over study [37]. Research integrity assessment (RIA) of the four included randomised trials [19, 31, 36, 39] are summarised in Additional file 5 [19, 31, 36, 39]—we identified “some concerns” for three trials [31, 36, 39].

**Table 1:** Key characteristics of included studies

Author, year	Study design	Country	Sample size included in analysis	GA of participants at time of sample collection (weeks)	Sample	Test and culture medium	Location of self-collection	GBS prevalence on self-collection	GBS prevalence on provider-collection	Funding sources
Arya 2008 [17]	Cross-sectional study	Ireland	600	35–37	Ano-vaginal swabs	Culture: enriched culture medium	Clinic	9.80%	11.00%	Not documented
Camus 2021 [31]	Non-inferiority randomised cross-over trial	France	224	7–41	Vaginal swabs	Culture: direct plating only	Clinic	8.90%	8.00%	Material or financial support from company
Chen 2021 [32]	Cross-sectional study	China	520	35–40	Rectovaginal swabs	Culture: enriched culture medium	Clinic	4.80%	3.45%	No conflicts of interest reported
Mercer 1995 [20] and Taylor 1997 [38]	Cross-sectional study	US	251	24- 42	Vaginal and rectal swabs	Culture: enriched culture medium	Clinic	17.50%	13.50%	Not documented
Molnar 1997 [33]	Cross-sectional study	Canada	163	26–28	Vaginal-anorectal swabs	Culture: enriched culture medium	Clinic	23.31%	19.63%	Not documented
Nebreda-Martin 2022 [34]	Cross-sectional study	Spain	189	35–37	Vaginal-rectal swab	Culture: direct plating only (71% of samples); enriched culture medium (29% of samples)	Home	13.61%	14.08%	No conflicts of interest reported
Price 2006 [19]	Randomised cross-over trial	Canada	330	35–37	Vaginal-rectal swab	Culture: enriched culture medium	Clinic	17.00%	18.80%	No conflicts of interest reported
Salvesen 1999 [35]	Cross-sectional study	Norway	80	> 37	Vaginal-anorectal swabs	Culture: enriched culture medium	Clinic	33.00%	21.00%	Not documented
Seto 2019 [36]	Randomised cross-over trial	Hong Kong	422	35–37	Vaginal and rectal swabs	Culture: enriched culture medium	Clinic	12.10%	19.20%	Not documented
Spieker 1999 [37]	Non-randomised cross-over study	US	240	28	Rectovaginal swabs	Culture: enriched culture medium	Clinic	7.90%	8.30%	Not documented
Torok 2000 [39]	Randomised cross-over trial	US	250	35–37	Anogenital swabs	Culture: enriched culture medium	Clinic	14.00%	16.00%	Not documented

No included studies which compared self-collected and provider-collected samples taken from different participants measured the outcome of maternal, perinatal, and/or neonatal outcomes, allowing comparison of risk of health outcomes between self-collection and provider-collection.

Included studies were published between 1995 and 2022 and were conducted in the US (3 studies) [20, 37–39], Canada (2 studies) [19, 33], Ireland (1 study) [17], Norway (1 study) [35], France (1 study) [31], Spain (1 study) [34], Hong Kong (1 study) [36], and China (1 study) [32]. All were studies of pregnant participants only, except for one study [31] in which 224/1027 participants (21.8%) were pregnant. Seven studies screened participants for GBS from 35 weeks’ GA [17, 19, 32, 34–36, 39], one French study screened participants from 7 to 41 weeks [31], one Canadian study screened participants from 26 to 28 weeks [33], and two US studies screened participants from 24 to 42 weeks [20, 38] and at 28 weeks [37].

The delay between self-sampling and provider sampling was short, with all self-collected and provider-collected swabs taken on the same day, except for one study [32], where samples may have been taken at different visits. In the four randomised cross-over trials [19, 31, 36, 39], participants were randomised to two groups, determining the order in which self-collected and provider-collected swabs were taken, with the comparative group’s swabs taken in the reverse order. One study [37] employed a similar cross-over design with participants split into two groups, but participant allocation was not randomised.

All studies tested for GBS colonisation with swab culture. The same test was performed on both self-collected and provider-collected samples. All sample collection involved either vaginal and rectal swabs [20, 36, 38], combined rectovaginal swabs [17, 19, 32, 34, 35, 37, 39], or vaginal swabs only [31]. Nine studies [17, 19, 20, 32, 33, 35–39] incubated specimens in enriched culture media (with or without direct plating), one study [31] used direct plating only, and one study [34] used direct plating only for 70.9% (*n* = 134/189) of sample pairs and enriched culture media for the remaining 29.1% (*n* = 55/189). The threshold for a positive test result was defined by authors as isolation of GBS on culture of either a combined vaginal-rectal swab, a vaginal swab only, or a rectal swab only.

In all studies, participants were given self-collection instructions during an in-person consultation. These instructions were verbal [17, 20, 35, 38], written or schematic [19, 31, 33, 34, 37, 39], verbal and written [32], or via video [36]. All self-collection occurred at the study clinics [17, 19, 20, 31, 32, 35–39], except for one study [34] in which self-collection occurred at home. In this study, participants were recruited at 30–32 weeks’ GA and instructed to undertake self-collection on the same morning as their 35–37-week antenatal consultation and bring their sample to their appointment, during which provider-sampling would occur.

The overall methodological quality of the included studies was generally poor (Table 2, Additional file 6 [17, 19, 20, 31–39]). No studies were at low risk of bias for all domains, though several studies did not report sufficient detail for some domains. There were no concerns regarding applicability, i.e. the extent to which included studies answered the review question.

**Table 2:** Risk of bias assessment of the included studies

**Studies**	**QUADAS-2 domains**						
	**Risk of bias**				**Concerns regarding applicability**		
	**Patient selection**	**Index test**	**Reference standard**	**Flow and timing**	**Patient selection**	**Index test**	**Reference standard**
Arya 2008 [17]	Unclear	Low	Low	Low	Low	Low	Low
Camus 2021 [31]	Unclear	High	Unclear	Low	Low	Low	Low
Chen 2021 [32]	Unclear	Low	Low	High	Low	Low	Low
Mercer 1995 [20] and Taylor 1997 [38]	Unclear	High	High	Low	Low	Low	Low
Molnar 1997 [33]	Unclear	High	Low	Low	Low	Low	Low
Nebreda-Martin 2022 [34]	Low	High	Low	High	Low	Low	Low
Price 2006 [19]	Low	Unclear	Unclear	Low	Low	Low	Low
Salvesen 1999 [35]	Unclear	High	High	High	Low	Low	Low
Seto 2019 [36]	Unclear	Low	Low	High	Low	Low	Low
Spieker 1999 [37]	Unclear	Low	Low	Low	Low	Low	Low
Torok 2000 [39]	High	Low	Low	Low	Low	Low	Low

### Sensitivity and specificity

**Fig. 2:**
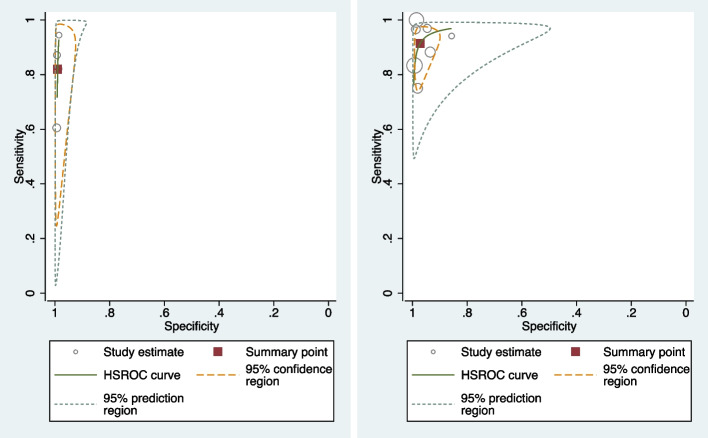
HSROC plots of randomised (left) and non-randomised (right) GBS studies that compared self-collection to provider-collection: The circles represent individual study estimates, with circle size proportional to sample size of each study. The continuous green line is the summary curve from the model. The red square represents the summary estimate for sensitivity and specificity and the yellow dotted line represents the 95% confidence region for this summary estimate. The green dotted line represents the 95% prediction region

**Fig. 3:**
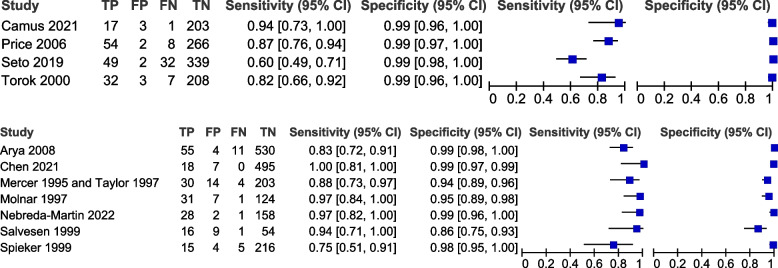
Forest plots of randomised (top) and non-randomised (bottom) studies that compare self-collected samples to provider-collected samples for GBS testing

Table 3 shows the pooled sensitivity (Se), specificity (Sp), diagnostic odds ratio (DOR), positive and negative likelihood ratios (LR + and LR −), and the inverse of the negative likelihood ratio (1/LR −) for the eleven included studies [17, 19, 20, 31–39]. Figure 2 displays the HSROC curves of the eleven studies. Figure 3 displays forest plots of the accuracy of GBS self-collection compared to provider-collection. We analysed randomised trials (4 studies, 1226 participants) and non-randomised studies (7 studies, 2043 participants) separately. Among the four randomised trials, pooled sensitivity was 82% (95% CI: 66–91%) with point estimates ranging from 60 to 94%, and pooled specificity was 99% (95% CI: 98–99%) with point estimates of 99%. In the seven non-randomised studies, pooled sensitivity was 91% (95% CI: 83–96%) with point estimates ranging from 75 to 100%, and pooled specificity was 97% (95% CI: 94–99%) with point estimates ranging from 86 to 100%.

**Table 3:** Pooled sensitivity and specificity from randomised (left) and non-randomised (right) studies that compared self-collected to provider-collected samples for GBS testing

Randomised studies (4 studies, 1226 participants)					Non-randomised studies (7 studies, 2043 participants)			
	**Coef**	**Std. err**	**95% conf. interval**		**Coef**	**Std. err**	**95% conf. interval**	
**Se**	0.82	0.06	0.66	0.91	0.91	0.03	0.83	0.96
**Sp**	0.99	0.00	0.98	0.99	0.97	0.01	0.94	0.99
**DOR**	458.48	212.39	184.93	1136.69	373.86	179.32	146.03	957.15
**LR + **	83.46	27.06	44.21	157.57	33.11	12.76	15.56	70.45
**LR − **	0.18	0.06	0.09	0.36	0.09	0.03	0.04	0.18
**1/LR − **	5.49	1.92	2.77	10.90	11.29	3.95	5.68	22.43

On visual inspection of forest plots (Fig. 3) to compare studies that could have been part of subgroups, no obvious trends were observed.

In the randomised trials, heterogeneity for sensitivity (68.85%) was higher than that of specificity (12.08%), and for non-randomised studies, heterogeneity for specificity (72.50%) was higher than that of sensitivity (37.38%) (Table 4). Despite the high *I*^2^ for sensitivity and specificity, the generalised between-study *I*^2^ was close to zero, which can be attributed to the correlation between sensitivity and specificity (rho =  − 1.00 and − 0.51) [40].

**Table 4:** Between-study heterogeneity statistics for randomised (left) and non-randomised (right) GBS studies that compare self-collection to provider-collection

	**Randomised studies** **(4 studies, 1226 participants)**	**Non-randomised studies** **(7 studies, 2043 participants)**
**rho**	− 1.00	− 0.51
	***I***^**2**^**(%)**	***I***^**2**^**(%)**
**Generalised**	0.03	51.93
**Se**	68.85	37.38
**Sp**	12.08	72.50

Sensitivity analyses are detailed in Additional file 7 [20, 33, 34, 37, 38].

Positive test prevalence is displayed in Table 1. A summary of all other additional outcomes is detailed in Additional file 8 [17, 19, 20, 31–39].

## Discussion

### Key findings

This review sought to determine whether self-collected samples are as accurate as provider-collected samples for the same test for detection of GBS colonisation in pregnant individuals. We found a limited evidence base (11 studies), there were possible integrity concerns for three trials, and no study was assessed as low risk of bias overall. Hence, it is possible that further high-quality research may draw different conclusions. We pooled eleven studies (3269 paired samples) and found generally high accuracy of self-collected compared to provider-collected samples for GBS detection. No study reported on whether a strategy of self-collection compared to provider-collection has an effect on maternal and perinatal health outcomes.

Meta-analysis of eleven studies found sensitivities of 82% (95% CI: 66–91%) and 91% (95% CI: 83–96%) and specificities of 99% (95% CI: 98–99%) and 97% (95% CI: 94–99%) for self-collection of samples for GBS testing in randomised trials and non-randomised studies, respectively. Individual point estimates were similar, with overlapping 95% CIs for sensitivity and specificity in most studies. The overall high sensitivity and specificity of self-collected swabs compared to provider-collected swabs supports the use of self-collection of samples for detection of GBS colonisation. However, among the eleven studies included in our analysis, particularly the four trials, the overall results indicate acceptable specificity, but lower sensitivity, with a wide 95% CI for the sensitivity summary estimate for trials (66–91%).

In ten studies [17, 19, 20, 31–33, 35–39], self-collection occurred in the clinic. In the one study [34] in which self-collection occurred at home, 3 to 7 weeks after receiving instructions, 27.3% (*n* = 52/190) of participants reported difficulties with self-collection. In that study, provider-collection occurred on the same day as self-collection after participants brought their self-collected samples to the clinic. However, the sensitivity and specificity point estimates were still high—Se 97% (95% CI: 82–100%), Sp 99% (95% CI: 96–100%). A self-sampling strategy will allow individuals to collect their own samples at a convenient time and location. Whilst the results from this study using self-collection outside of the clinic setting are promising, further confirmatory studies are needed.

In one study [36], the majority of participants (66.6%, *n* = 273/410) reported difficulty with self-collection, despite written information sheets and a short instructional video. This study had the lowest sensitivity point estimate of the included studies (Se 60% [95% CI: 49–71%], Sp 99% [98% CI: 96–100%]). Whilst all studies provided information to participants on self-collection procedures, this information was not standardised nor assessed for participant understanding—this is a potential source of variation in accuracy of self-collection. There may be a role for guidelines that provide standardised self-collection instructions and processes. In the studies that reported on self-collection uptake, a proportion of eligible participants declined the option, and in the studies that reported on sampling preference, there was even split between those favouring provider-collection or self-collection. This highlights the importance of allowing availability of options, including self-care interventions, to expand patient choice and autonomy.

### Strengths and limitations

To our knowledge, this is the first systematic review on self-collection of samples for RTI testing, including GBS, in pregnant individuals. Strengths of this review involved searching a large number of databases without language or publication date limitations, and sourcing additional outcome data through contacting study investigators. We embedded a novel adapted RIA tool, which identified that some of the included trials could be problematic. We did not exclude these trials on this basis alone, requested further data from triallists, and reported all RIA domains for transparency. All included studies made direct within-study comparisons, by performing the same test on self-collected and provider-collected samples from all participants. Hence, each participant acted as their own control and studies were thus less prone to bias due to confounding [26]. This review nonetheless has some limitations. One possible limitation is that we may not have identified studies of primarily non-pregnant patients that may have included a subpopulation of pregnant individuals. To mitigate this, we sought support from a librarian for our search strategy, and we screened full texts for any mention of pregnancy, even when the title or abstract did not suggest it.

### Implications for policy, clinical practice, and future research

Our findings support offering the choice of sample self-collection for GBS detection in antenatal care settings, particularly given the high sensitivity and specificity of self-collected compared to provider-collected samples, and the finding that many people accept (and even prefer) this approach. However, given the limited evidence base, with all studies at high or unclear risk of bias, caution must be taken when interpreting the meta-analysis findings of sensitivity and specificity. No studies were found on the effect of self-collection on maternal, perinatal, or neonatal health outcomes, so we could not explore whether this strategy is better or worse in terms of pregnancy-related outcomes. WHO guidelines note that large multicentre trials are needed to evaluate the effects GBS screening and confirm whether screening reduces preterm birth and perinatal mortality in low- and middle-income countries [41].

## Conclusions

Expanding GBS screening and task-shifting away from clinical services to home-based and community service testing could expand access and decrease burden on healthcare systems. This is particularly so in limited-resource settings with healthcare infrastructure limitations, although sample processing is dependent on the availability of laboratory services and trained staff, which may not be feasible in all low-income country settings. Nearly all studies we identified were conducted in high-income countries; self-collection could be useful for individuals in rural and remote regions in high-income countries if the option of posting samples was available. The evidence from this meta-analysis supports the option of self-collection of samples for GBS testing for individuals who decline provider-collection, who are hard to reach, or face barriers to antenatal care. Availability of accurate sampling options for screening to suit patient choice can hopefully improve uptake of GBS testing and thus reduce the incidence of EOGBS. In high-income settings with universal GBS screening in pregnancy, prevalence of adherence to antenatal GBS screening has been reported as ranging from 52 to 85.5% in Australian and US studies [42–44].

Whilst sample self-collection, as an additional option to provider-collection for detection of GBS colonisation, is promising, further research is required to determine whether self-collection at home, without same-day instructions on how to self-collect, would be as accurate, and improve uptake.

### Supplementary Information

**Additional file 1. **PRISMA Checklist.

**Additional file 2. **Literature retrieval strings

**Additional file 3. **Outcomes.

**Additional file 4. **Studies excluded at full-text screening stage

**Additional file 5: Table S1.** Research/trial integrity assessment

**Additional file 6: Figure S1.** Risk of bias assessment and applicability of the included studies.

**Additional file 7. **Sensitivity analyses.

**Additional file 8: Table S2.** Summary of additional outcomes.

## Data Availability

All data generated or analysed during this study are included in this published article and its supplementary information files.

## Abbreviations

CI: Confidence interval
DOR: Diagnostic odds ratio
EOGBS: Early-onset Group B Streptococcal disease
FN: False negatives
FP: False positives
GA: Gestational age
GBS: Group B streptococcus
HSROC: Hierarchical summary receiver operating characteristic
HPV: Human papillomavirus
IAP: Intrapartum antibiotic prophylaxis
LR +: Positive likelihood ratio
LR -: Negative likelihood ratio
NAAT: Nucleic acid amplification test
PRISMA: Preferred Reporting Items for Systematic Reviews and Meta-Analyses
RTI: Reproductive tract infection
Se: Sensitivity
Sp: Specificity
STI: Sexually transmitted infection
TN: True negatives
TP: True positives
UK: United Kingdom
US: United States
WHO: World Health Organization

## References

[1] Seale AC, Bianchi-Jassir F, Russell NJ (2017). Estimates of the burden of group B streptococcal disease worldwide for pregnant women, stillbirths, and children. Clin Infect Dis..

[2] ACOG (2020). Prevention of group B streptococcal early-onset disease in newborns: ACOG Committee Opinion, Number 797. Obstet Gynecol..

[3] Russell NJ, Seale AC, O’Driscoll M, et al. Maternal colonization with group B streptococcus and serotype distribution worldwide: systematic review and meta-analyses. Clin Infect Dis. 2017;65(suppl_2):S100–11. 10.1093/cid/cix658 PMC5848259 29117327

[4] World Health Organization (2015). WHO recommendations for prevention and treatment of maternal peripartum infections.

[5] Hasperhoven GF, Al-Nasiry S, Bekker V, Villamor E, Kramer B (2020). Universal screening versus risk-based protocols for antibiotic prophylaxis during childbirth to prevent early-onset group B streptococcal disease: a systematic review and meta-analysis. BJOG.

[6] Procter SR, Gonçalves BP, Paul P (2023). Maternal immunisation against group B Streptococcus: a global analysis of health impact and cost-effectiveness. PLoS Med.

[7] Virranniemi M, Raudaskoski T, Haapsamo M (2019). The effect of screening-to-labor interval on the sensitivity of late-pregnancy culture in the prediction of group B streptococcus colonization at labor: a prospective multicenter cohort study. Acta Obstet Gynecol Scand.

[8] Daniels J, Gray J, Pattison H (2009). Rapid testing for group B streptococcus during labour: a test accuracy study with evaluation of acceptability and cost-effectiveness. Health Technol Assess.

[9] Verani J, McGee L, Schrag S (2010). Prevention of perinatal group B streptococcal disease–revised guidelines from CDC, 2010. MMWR Recomm Rep.

[10] World Health Organization (2021). WHO guideline on self-care interventions for health and well-being.

[11] Ogale Y, Yeh PT, Kennedy CE (2019). Self-collection of samples as an additional approach to deliver testing services for sexually transmitted infections: a systematic review and metaanalysis. BMJ Glob Health.

[12] Lunny C, Taylor D, Hoang L (2015). Self-collected versus clinician-collected sampling for chlamydia and gonorrhea screening: a systemic review and meta-analysis. PLoS ONE.

[13] Arbyn M, Smith SB, Temin S, Sultana F, Castle P (2018). Detecting cervical precancer and reaching underscreened women by using HPV testing on self samples: updated meta-analyses. BMJ.

[14] Paudyal P, Llewellyn C, Lau J, Mahmud M, Smith H. Obtaining self-samples to diagnose curable sexually transmitted infections: a systematic review of patients’ experiences. PLoS ONE. 2015;10(4):e0124310. 10.1371/journal.pone.0124310 PMC4409059 25909508

[15] Kpokiri E, Marley G, Tang W (2020). Diagnostic infectious diseases testing outside clinics: a global systematic review and meta-analysis. Open Forum Infect Dis..

[16] Fajardo-Bernal L, Aponte-Gonzalez J, Vigil P (2015). Home-based versus clinic-based specimen collection in the management of Chlamydia trachomatis and Neisseria gonorrhoeae infections. Cochrane Database of Syst Rev..

[17] Arya A, Cryan B, O’Sullivan K, Greene RA, Higgins JR. Self-collected versus health professional-collected genital swabs to identify the prevalence of group B streptococcus: a comparison of patient preference and efficacy. Eur J Obstet Gynecol Reprod Biol. 2008;139(1):43–5. 10.1016/j.ejogrb.2007.12.005 18255214

[18] Hicks P, Diaz-Perez MJ (2009). Patient self-collection of group B streptococcal specimens during pregnancy. J Am Board Fam Med.

[19] Price D, Shaw E, Howard M, Zazulak J, Waters H, Kaczorowski J (2006). Self-sampling for group B streptococcus in women 35 to 37 weeks pregnant is accurate and acceptable: a randomized cross-over trial. J Obstet Gynaecol Can.

[20] Mercer BM, Taylor MC, Fricke JL, Baselski VS, Sibai BM (1995). The accuracy and patient preference for self-collected group B Streptococcus cultures. Am J Obstet Gynecol.

[21] Narasimhan M, Logie CH, Gauntley A, et al. Self-care interventions for sexual and reproductive health and rights for advancing universal health coverage. Sex Reprod Health Matters. 2020;28(2):298–314. 10.1080/26410397.2020.1778610 PMC7887951 32530386

[22] McInnes MDF, Moher D, Thombs BD (2018). Preferred reporting items for a systematic review and meta-analysis of diagnostic test accuracy studies: the PRISMA-DTA statement. JAMA.

[23] Weibel S, Popp M, Reis S, Skoetz N, Garner P, Sydenham E. Research Integrity Assessment (RIA) Tool for RCTs in evidence synthesis. Zenodo. 2022. Available from: 10.5281/zenodo.7024699. Accessed 1 Oct 2023. 10.1002/jrsm.1599 PMC10551123 36054583

[24] Weibel S, Popp M, Reis S, Skoetz N, Garner P, Sydenham E. Identifying and managing problematic trials: a research integrity assessment tool for randomized controlled trials in evidence synthesis. Res Syn Meth. 2022:14(3):357–69. 10.1002/jrsm.1599 PMC10551123 36054583

[25] Whiting PF, Rutjes AW, Westwood ME (2011). QUADAS-2: a revised tool for the quality assessment of diagnostic accuracy studies. Ann Intern Med..

[26] Cochrane Collaboration. Cochrane Handbook for Systematic Reviews of Diagnostic Test Accuracy, Version 2.0, 2022. Birmingham: Cochrane Collaboration; 2022. Available from: https://training.cochrane.org/handbook-diagnostic-test-accuracy. Accessed 1 Sept 2023.

[27] Harbord RM, Whiting P (2009). metandi: Meta-analysis of diagnostic accuracy using hierarchical logistic regression. Stata J.

[28] Takwoingi Y, Guo B, Riley RD, Deeks JJ (2017). Performance of methods for meta-analysis of diagnostic test accuracy with few studies or sparse data. Stat Methods Med Res.

[29] Zhou Y, Dendukuri N (2014). Statistics for quantifying heterogeneity in univariate and bivariate meta-analyses of binary data: the case of meta-analyses of diagnostic accuracy. Stat Med.

[30] Page MJ, McKenzie JE, Bossuyt PM (2021). The PRISMA 2020 statement: an updated guideline for reporting systematic reviews. BMJ.

[31] Camus C, Penaranda G, Khiri H (2021). Acceptability and efficacy of vaginal self-sampling for genital infection and bacterial vaginosis: a cross-sectional study. PLoS ONE.

[32] Chen R, Wu L, Ma F, Chen X, Zhu Y. The accuracy and influencing factors for preference of self-sampling in group B streptococcus screening: a cross-sectional study. J Matern Fetal Neonatal Med. 2021:35(25):5194–8. 10.1080/14767058.2021.1875441 33618588

[33] Molnar P, Biringer A, McGeer A, McIsaac W (1997). Can pregnant women obtain their own specimens for group B streptococcus? A comparison of maternal versus physician screening. The Mount Sinai GBS Screening Group. Fam Pract..

[34] Nebreda-Martin L, Albisu-Del Campo A, Valle-Ruiz de Larrea L, Gonzalez-Rodriguez G, Arana-Arri E, Paz-Pascual C (2022). Effectiveness of the vagino-rectal exudate self-sampling for prenatal screening of Streptococcus agalactiae infection. GALL study]. Efectividad de la autotoma del exudado vaginorrectal para el cribado prenatal de la infeccion por Streptococcus agalactiae Estudio GALL. Aten Primaria..

[35] Salvesen KA, Dahlo R, Sommer T, Bevanger L (1999). [Pregnant women themselves can take specimens for identification of group B streptococci carriers]. Gravide kan selv ta prover for pavisning av baerertilstand for gruppe B-streptokokker. Tidsskr Nor Laegeforen..

[36] Seto MTY, Ko JKY, Cheung KW (2019). The accuracy of self-screening of group B streptococcus in pregnant women-a randomized crossover study. J Obstet Gynaecol Can.

[37] Spieker MR, White DG, Quist BK (1999). Self-collection of group B Streptococcus cultures in pregnant women. Mil Med.

[38] Taylor MC, Mercer BM, Engelhardt KF, Fricke JL (1997). Patient preference for self-collected cultures for group B streptococcus in pregnancy. J Nurse Midwifery.

[39] Torok PG, Dunn JR (2000). Self-collection of antepartum anogenital group B streptococcus cultures. J Am Board Fam Pract.

[40] Nyaga VN, Arbyn M (2022). Metadta: a Stata command for meta-analysis and meta-regression of diagnostic test accuracy data – a tutorial. Arch Public Health.

[41] World Health Organization (2016). WHO recommendations on antenatal care for a positive pregnancy experience.

[42] Pangerl S, Sundin D, Geraghty S (2022). Adherence to screening and management guidelines of maternal group B Streptococcus colonization in pregnancy. J Adv Nurs.

[43] Santillan DA, Hubb AJ, Nishimura TE (2022). Group B streptococcus screening and treatment adherence in pregnancy: a retrospective cohort study and opportunities for improvement. AJPM Focus.

[44] Schrag SJ, Arnold KE, Mohle-Boetani JC (2003). Prenatal screening for infectious diseases and opportunities for prevention. Obstet Gynecol.

