# Supplementary material for: Self-collection of samples for group B streptococcus testing during pregnancy: a systematic review and meta-analysis
Source: BMC Med. 2023 Dec 18;21:498. doi: 10.1186/s12916-023-03186-x (PMC10729404; doi:10.1186/s12916-023-03186-x)
Supplement: Supplementary file 2 — Additional file 2. Literature retrieval strings [file 12916_2023_3186_MOESM2_ESM.docx]

## Additional file 2: Literature retrieval strings

1. **Concept A – Self-care**

**Search terms:** self care, home based care, self exam, home based exam, self administer, home administration, self use, self usage, self test, home based test, home based kit, self sample, home based sampling, self screen, home based screen, self diagnosis, home based diagnosis, self collection, home based collection, self measurement, home based measurement, self obtain, home based obtainment, self assessment, home based assessment, self evaluation, home based evaluation, diagnostic self evaluation, patient collection, individual collection

| **CINAHL subject thesaurus** | (MH "Self-Management") OR (MH “Self-testing”) OR (MH "Self-Diagnosis+") |
| --- | --- |
| **Medline/EBM (MeSH)** | self-care/ or self-examination/ or self-testing/ or self-assessment/ or diagnostic self evaluation/ or self administration/ |
| **EMBASE (MeSH)** | self care/ or self-examination/ or self-testing/ or self evaluation/ |
| **Textwords (CINAHL)** | (self N3 (care or manag* or exam* or administ* or us* or test* or screen* or diagnos* or collect* or measure* or obtain* or monitor* or assess* or evaluat*))  or  (patient-cent* N2 care)  or  (person-cent* N2 care)  or (“patient-orient*” N2 care)  or  (home based N2 (kit* or exam* or administ* or or diagnos* or obtain* or assessment* or evaluat*))  or  (home N2 (kit* or exam* or administ or diagnos* or obtain* or assessment* or evaluat*))  or  individual* collect*  or  patient collect* |
| **Text words (Medline/EBM/EMBASE)** | (self adj (care or exam* or administ* or "use" or "useage" or "usage" or test* or sampl* or screen* or diagnos* or collect* or measur* or obtain* or assess* or evaluat*)).mp.  or  (selfcare or selfexam* or selfadminist* or selfuse* or selfusage or selftest*or selfsampl* or selfscreen* or selfdiagnos* or selfcollect* or selfmeasu* or selfobtain* or selfassess or selfevaluat*).mp.  or  (((patient or individual) adj collect*) not collective*).mp.  or  ((home or "home-based" or homebased) adj4 (care or exam* or test* or kits or kit or sampl* or screen* or diagnos* or collect* or measur* or obtain* or assess*)).mp.  or  ((home or "home-based" or homebased) adj3 evaluat*).mp.  or  (((home or "home-based" or home based) adj4 administ*) not administrator*).mp. |
| **Text words (Maternity and Infant care)** | (self-care or selfcare or ((home or "home-base*" or homebase*) adj4 care) or self-exam* or selfexam* or ((home or "home-base*" or homebase*) adj4 exam*) or self-administ* or selfadminist* or ((home or "home-base*" or home base*) adj4 administ*) or self-use or selfuse or self-usage or selfusage or selftest*or self-test* or ((home or "home-base*" or homebase*) adj4 (test* or kits or kit)) or self-sampl* or selfsampl* or ((home or "home-base*" or homebase*) adj4 sampl*) or self-screen* or selfscreen* or ((home or "home-base*" or homebase*) adj4 screen*) or self-diagnos* or selfdiagnos* or ((home or "home-base*" or homebase*) adj4 diagnos*) or self-collect* or selfcollect* or ((home or "home-base*" or homebase*) adj4 collect*) or self-measur* or selfmeasu* or ((home or "home-base*" or homebase*) adj4 measur*) or self-obtain* or selfobtain* or ((home or "home-base*" or homebase*) adj4 obtain*) or self-assess* or selfassess or ((home or "home-base*" or homebase*) adj4 assess*) or self-evaluat* or selfevaluat* or "diagnostic self evaluat*" or "diagnosticself evaluat*" or "diagnostic selfevaluat*" or ((home or "home-base*" or homebase*) adj3 evaluat*) or "patient-collect*" or patientcollect* or "individual collect*" or individualcollect*).ti,ab,ss,sx,de,hw. |

1. **Concept B – Pregnant individuals**

**Subject terms:** prenatal, antenatal, prenatal diagnosis, prenatal care, prenatal healthcare, perinatal, perinatal diagnosis perinatal care, perinatal healthcare, intrapartum, intrapartum care, intrapartum diagnosis, pregnancy care, pregnancy healthcare, pregnancy outcome, pregnancy complications, maternal care, maternal outcome, maternal complication, obstetric care, obstetric healthcare, obstetric outcome, obstetric complication, gravidity, nulligravid, nulliparous, primigrav, primipara, multigravida, multipara, parous, pregnant, pregnancy, pregnancy health service, obstetric health service, maternal health service, obstetric delivery, first trimester, second trimester, third trimester, pregnant women, pregnant woman, gestation, expectant mother, expectant women, expectant female, pregnancy in adolescent, natal

| **CINAHL subject thesaurus** | (MH "Expectant Mothers") OR (MH "Pregnancy Trimesters") OR (MH "Pregnancy Outcomes") OR (MH "Pregnancy, Multiple") OR (MH "Pregnancy Trimesters") OR (MH "Perinatal Period") OR (MH "Childbirth") OR (MH "Periconceptual Period") OR (MH "Prenatal Care") OR (MH "Prepregnancy Care") |
| --- | --- |
| **Medline/EBM (MeSH)** | gravidity/ or pregnancy/ or pregnancy in adolescence/ or pregnancy outcome/ or abortion, spontaneous/ or live birth/ or stillbirth/ or pregnancy, high-risk/ or pregnancy complications/ or pregnancy complications, infectious/ or puerperal Infection/ or perinatal care/ or prenatal care/ or maternal health services/ or delivery, obstetric/ or abortion, spontaneous/ or abortion, habitual/ or abortion, incomplete/ or abortion, missed/ or abortion, septic/ or embryo loss/ or abortion, threatened/ or pregnancy trimesters/ or pregnancy trimester, first/ or pregnancy trimester, second/ or pregnancy trimester, third/ or prenatal diagnosis/ |
| **EMBASE (MeSH)** | gravidity/ or pregnancy/ or adolescent pregnancy/ or first trimester pregnancy/ or second trimester pregnancy/ or third trimester pregnancy/ or spontaneous abortion/ or pregnancy  disorder/ or pregnancy complication/ or pregnancy/ or pregnancy outcome/ or high risk pregnancy/ or live birth/ or stillbirth/ or puerperal infection/ or perinatal care/ or prenatal care/ or prenatal diagnosis/ or prenatal screening/ or maternal health service/ or maternal care/ or obstetric delivery/ or exp abortion/ |
| **Textwords (CINAHL)** | Prenatal or antenatal or perinatal or intrapartum or pregnan* or matern* or obstetric* or gravidit* or nulligravid* or nullipara*, primigrav* or primipara* or multigravid* or multipar* or parous or trimester* or gestation or (expectant adj (mother* or wom#n or female*) or natal or preconception or birth* or childbirth* or labour* or labor* |
| **Text words (Medline/EBM/EMBASE)** | (natal or antenatal* or prenatal* or perinatal or intrapartum or intra-partum or pregnan* or maternal care or maternal healthcare or maternal health care or maternal health service or maternal outcom* or maternal complication* or obstetric* or gravid* or nulligrav* or nullipar* or primigrav* or primipar* or multigrav* or multipar* or trimester* or gestation*).mp.  or  ((expectant or expecting or gestating) adj (mother* or wom#n or female* or girl*)).mp. |
| **Text words (Maternity and Infant care)** | (natal or antenatal* or "ante natal" or prenatal* or "pre-natal" or perinatal* or "peri-natal" or intrapartum* or "intra-partum" or "pregnancy care" or pregnancycare or pregnancyhealth* or "pregnancy health*" or (pregnancy adj3 outcom*) or pregnancyoutcom* or (pregnancy adj3 complication*) or pregnancycomplication* or "maternal care" or maternalcare or "maternal health*" or maternalhealth* or (maternal adj3 outcom*) or maternaloutcom* or (maternal adj3 complication*) or maternalcomp* or "obstetric* care*" or obstetriccare* or "obstetric health*" or obstetrichealth* or (obstetric* adj3 outcom*) or obstetricoutcome* or (obstetric* adj3 complication*) or obstetriccomp* or gravid* or nulligrav* or nullipar* or primigrav* or primipar* or multigrav* or multipar* or pregnan* or trimester or gestation* or (expectant adj (mother* or wom#n or female*))).ti,ab,sx,ss,de,hw. |

1. **Concept C – Reproductive Tract Infections**

**Subject terms:** reproductive tract infection, genital tract infection, genital system infection, reproductive system infection,  venereal disease, sexually transmitted infection, sexually transmitted disease, bacterial sexually transmitted infection, sexually transmissable infection, sexually transmissable disease, STD, STI,  chlamydia, chlamydia infection, chlamydia trachomatis, gonorrhoea, gonococcal, gonococci, nesisseria gonorrhoaea, gono?r*ea, trichomonas, trichiomiasis, trichomonas infection, trichomonas vaginitis, trichomonas vagininalis, trichomona* trichomonias*, anaerobic vaginositis, non-specific vaginitis, bacterial vaginosis, vaginal bacteriosis, gardnerella vaginitis, gardneralla vaginalis, vaginal yeast infection, vaginal thrush, candidal vulvovaginitis, vaginal candidia, vulvovaginal candidiasis, monilial vaginitis, monilia, vulvovaginal monilia,  vaginal monilia, human papilloma virus, HPV, group B strep infection, GBS, GBS infection, group B strep, group B streptococcous, group B streptococcous infection, group B streptococcal infection

| **CINAHL subject thesaurus** | (MH "Chlamydia") OR (MH "Chlamydia Infections") OR (MH "Chlamydia Trachomatis") OR (MH "Trichomonas Vaginitis") OR (MM "Trichomonas Infections") OR (MH "Gonorrhea") OR (MH "Vaginosis, Bacterial") OR (MH “Vaginitis”) OR (MH "Candidiasis, Vulvovaginal") OR (MH "Papillomaviruses") |
| --- | --- |
| **Medline/EBM (MeSH)** | reproductive tract infections/ or sexually transmitted diseases/ or sexually transmitted diseases, bacterial/ or chlamydia infections/ or chlamydia/ or chlamydia trachomatis/ or gonorrhea/ or neisseria gonorrhoeae/ or trichomonas infections/ or trichomonas/ or trichomonas vaginitis/ or trichomonas vaginalis/ or candidiasis, vulvovaginal/ or Candida albicans/ or gardnerella/ or gardnerella vaginalis/ or vaginosis, bacterial/ or vaginitis/ or vulvovaginitis/ or human papillomavirus / or human papillomavirus / or human papillomavirus / or human papillomavirus / or human papillomavirus / |
| **EMBASE (MeSH)** | genital tract infection/ or female genital tract infection/ or sexually transmitted disease/ or chlamydiasis/ or chlamydia trachomatis/ or gonorrhea/ or Neisseria gonorrhoeae/ or trichomoniasis/ or vaginal trichomoniasis/ or Trichomonas vaginalis/ or Trichomonas/ or Trichomonas vaginalis test kit/ or candidiasis/ or genital candidiasis/ or thrush/ or vagina candidiasis/ or Gardnerella vaginalis/ or Gardnerella/ or Gardnerella infection/ or vaginitis/ or vulvovaginitis/ or Wart virus/ |
| **Textwords (CINAHL)** | Reproductive tract infection* or RTI* or sexual* transmit* infect* or sexual* transmi* diseas* or STD* or STI* or chlamydia* or gonorr* or or trichomonas* or trichomoniasis* or bacterial vaginosis* or vaginositis* or vaginal yeast infection* or vaginal thrush* or candida vulvovaginitis* or vaginal candidiasis* or HPV or human papilloma virus or group B strep* infection* or GBS or group B stretococc* or genital tract infection* or genital system infection* or reproductive system infection* or venereal disease* or gonococc*or nesisseria or trichomonas or anaerobic vaginositis or vaginal bacteriosis or gardner#lla vaginalis or vaginal candidia* or vulvovaginal candidia* or monilia |
| **Text words (Medline/EBM/EMBASE)** | reproductive tract infection* or reproductive tractinfection* or genital tract infection* or genitaltract infection* or genital tractinfection* or genitaltractinfection* or genital system infection* or genitalsystem infection* or genital systeminfection* or genitalsysteminfection* or reproductive system infection* or reproductivesystem infection* or  reproductive systeminfection* or reproductivesysteminfection* or sexually transmi* infection* or sexuallytransmi*infection* or sexuallytransmi* infection* or sexually transmi*infection* or sexually transmi* disease* or sexuallytransmi*disease* or sexuallytransmi* disease* or sexually transmi*disease* or STD* or STI* or venereal disease* or venerealdisease* or chlamydia* or gono?r*ea* or gonococc* or trichomona* or trichomonias* or gardnerella or bacterial vagin* or bacterialvagin* or anaerobic vagin* or anaerobicvagin* or non-specific vagin* or non-specificvagin* or nonspecific vagin* or nonspecificvagin* or vagina* bacteriosis or vagina*bacteriosis or vagina* yeast infection* or vagina*yeastinfection* or vagina* yeastinfection* or vagina*yeast infection* or vagina* thrush or vagina*thrush or candida* vulvovaginitis or candida*vulvovaginitis or vagina* candidia* or vagina*candidia* or vulvovagina* candidia* or vulvovagina*candidia* or genital candidia* or genitalcandidia* or monilia* vagin* or monilia*vagin* or vulvovagina* monilia* or vulvovagina*monilia* or vagina* monilia* or vagina*monilia* or monilia* or human papilloma* or human papilloma* or HPV or group B strep* or groupB strep* or groupBstrep* or group Bstrep* or GBS.mp. |
| **Text words (Maternity and Infant care)** | ((reproductive* or genital* or sexual* or vereneal or vagina* or vulvovagina*) adj3 (disease* or infection* or thrush or candida* or candidia* or yeast or monili* or monilinia*) or tractinfect* or transmittedinfect* or transmitteddisease* or verenealinfect* or verealdisease* or vaginalinfect* or vaginaldisease* or "candida* vulvo*" or "candida*vulvo*" or "vulvovagina*candidia*" or "genitalcandidia*" or "vulvovagina*monili*" or "vagina*monili*" std or stds or sti or stis or chlamydia* or gono?r*ea* or gonococc* or trichomona* or trichomonias* or gardnerella or "bacteria* vagin*" or bacteria?vagin* or "anaerobic vagin*" or anaerobicvagin* or "non-specific vagin*" or "non-specificvagin*" or "nonspecific vagin*" or nonspecificvagin* or "vagina* bacteri" or "vagina*bacteri*" or "monilia* vagin*" or "monilia*vagin*" or papillomavirus* or "papilloma virus*" or humanpapilloma* or HPV or "group B strep*" or groupB* or "Bstrep*" or "GBS infect*" or GBSinfect* or GBS).ti,ab,sx,ss,de,hw. |
