# Supplementary material for: Self-collection of samples for group B streptococcus testing during pregnancy: a systematic review and meta-analysis
Source: BMC Med. 2023 Dec 18;21:498. doi: 10.1186/s12916-023-03186-x (PMC10729404; doi:10.1186/s12916-023-03186-x)
Supplement: Supplementary file 3 — Additional file 3. Outcomes. [file 12916_2023_3186_MOESM3_ESM.docx]

## Additional file 3: Outcomes

Primary outcomes:

1. Diagnostic accuracy (sensitivity and specificity)
2. Risk of poor maternal, perinatal, and/or neonatal outcomes, including: neonatal infection; neonatal death; ectopic pregnancy; miscarriage; preterm birth; premature rupture of membranes; low birthweight; stillbirth; perinatal and postpartum infection

Additional outcomes:

1. Uptake of self-collection of samples for GBS testing (e.g., the proportion who accepted and completed the test)
2. Sampling order (e.g., whether self-collection or provider-collection was performed first)
3. Proportion of people who tested positive for GBS colonisation (case finding)
4. Linkage to clinical assessment or treatment following a positive test result
5. Patient acceptability (e.g., if collection of the specimen is reported as comfortable and secure) – measured by patient satisfaction surveys and questionaries
6. Patient preference
7. Feasibility of self-collection of samples
