# Supplementary material for: Self-collection of samples for group B streptococcus testing during pregnancy: a systematic review and meta-analysis
Source: BMC Med. 2023 Dec 18;21:498. doi: 10.1186/s12916-023-03186-x (PMC10729404; doi:10.1186/s12916-023-03186-x)
Supplement: Supplementary file 4 — Additional file 4. Studies excluded at full-text screening stage [file 12916_2023_3186_MOESM4_ESM.docx]

## Additional file 4: Studies excluded at full-text screening stage

| **Author** | **Published Year** | **Title** | **Exclusion reason** |
| --- | --- | --- | --- |
| Saling | 1995 | [Initial results of “preventative self-care by pregnant patients” for prevention of premature labour] | F |
| Tabrizi | 1996 | Patient-administered tampon-collected genital cells in the assessment of Chlamydia trachomatis infection using polymerase chain reaction | A |
| Hordnes | 1998 | Self-sampled and air-dried cervicovaginal secretions can be used for analyses of mucosal antibodies | G |
| Hoyme | 1998 | Reduction of prematurity by pH-screening | E |
| Gray | 1998 | A randomized trial of STD control during pregnancy in Rakai, Uganda: impact on maternal and infant health | B |
| Ostergaard | 1999 | [DNA amplification in the diagnosis of urogenital Chlamydia trachomatis infection] | B |
| Chernesky | 1999 | Newly available and future laboratory tests for sexually transmitted diseases (STDs) other than HIV | B |
| Saling | 1999 | Prematurity-prevention program. Cooperation between the doctor, midwife and patient | F |
| Hoyme | 2000 | Bacterial vaginosis as risk factor | E |
| Saling | 2000 | [A new method for self assessment of the pH value of vaginal secretions using an indicator-coated panty liner--initial preliminary report] | E |
| Smith | 2001 | Self-obtained vaginal swabs for diagnosis of treatable sexually transmitted diseases in adolescent girls | A |
| vanValkengoed | 2001 | Cost effectiveness analysis of a population based screening programme for asymptomatic Chlamydia trachomatis infections in women by means of home obtained urine specimens | A |
| Pritzker | 2001 | Self-assessment in obstetrics and gynecology. Evaluation and management of vaginitis: review questions | B |
| Garrow | 2002 | The diagnosis of chlamydia, gonorrhoea, and trichomonas infections by self obtained low vaginal swabs, in remote northern Australian clinical practice | G |
| Hoyme | 2002 | Results and potential consequences of the thuringia prematurity preventional campaign 2000 | F |
| Oakeshott | 2002 | Detection of Chlamydia trachomatis infection in early pregnancy using self-administered vaginal swabs and first pass urines: a cross-sectional community-based survey | E |
| Serlin | 2002 | What sexually transmitted disease screening method does the adolescent prefer? Adolescents' attitudes toward first-void urine, self-collected vaginal swab, and pelvic examination | A |
| Andersen | 2002 | Diagnosis of urogenital chlamydia trachomatis infections by home-obtained, mailed samples: do we need a telephone hotline for information and advice? | A |
| Garcia | 2003 | Cross-sectional study of patient- and physician-collected cervical cytology and human papillomavirus | C |
| Yen | 2003 | Bacterial vaginosis in sexually experienced and non-sexually experienced young women entering the military | C |
| Boskey | 2004 | Acceptability of a self-sampling technique to collect vaginal smears for gram stain diagnosis of bacterial vaginosis | D |
| Heinke | 2004 | Self-testing of bacterial vaginosis to prevent premature births | B |
| Hoyme | 2004 | Efficient prematurity prevention is possible by pH-self measurement and immediate therapy of threatening ascending infection | F |
| Hoyme | 2004 | [Efficient prevention of prematurity – the Thuringian model] | F |
| Kahn | 2004 | Comparison of adolescent and young adult self-collected and clinician-collected samples for human papillomavirus | C |
| Novak | 2004 | A Swedish cost-effectiveness analysis of community-based Chlamydia trachomatis PCR testing of postal urine specimens obtained at home | A |
| Tebb | 2004 | Home STI testing: the adolescent female's opinion | A |
| Hay | 2005 | Non-invasive chlamydia testing of pregnant teenagers | D |
| Knesel | 2005 | Preliminary evaluation of a cervical self-sampling device with liquid-based cytology and multiparameter molecular testing | A |
| Prusty | 2005 | Human papillomavirus (HPV) DNA detection in self-collected urine | A |
| Saling | 2005 | [The lactobacilli-protection system of pregnant women—efficient prevention of premature births by early detection of disturbances] | F |
| Schachter | 2005 | Vaginal swabs are the specimens of choice when screening for Chlamydia trachomatis and Neisseria gonorrhoeae: results from a multicenter evaluation of the APTIMA assays for both infections | A |
| Siegmund-Schultze | 2005 | pH self assessment to reduce the risk of preterm birth - A pilot study becomes a model project | H |
| Bresson | 2006 | [Self-collected vaginal swabs to diagnosis bacterial vaginosis during pregnancy: A pilot study] | D |
| HolandaJr | 2006 | Primary screening for cervical cancer through self sampling | C |
| Rose | 2007 | Self-obtained vaginal swabs for PCR chlamydia testing: A practical alternative | J |
| Schleussner | 2007 | New self testing procedures: Femintim and BabySafe | B |
| Skidmore | 2007 | Testing for Chlamydia trachomatis: self-test or laboratory-based diagnosis? | B |
| Oakeshott | 2008 | Community-based trial of screening for Chlamydia trachomatis to prevent pelvic inflammatory disease: the POPI (prevention of pelvic infection) trial | C |
| Langille | 2008 | A pilot project for chlamydia screening in adolescent females using self-testing: characteristics of participants and non-participants | A |
| Libbus | 2008 | Chlamydia Rapid Test was moderately accurate for diagnosing Chlamydia infection in women | B |
| Rouse | 2008 | [Commentary on] Self-collected versus health professional-collected genital swabs to identify the prevalence of group B Streptococcus: a comparison of patient preference and efficacy | B |
| STD Quarterly | 2009 | Program launches STD at-home testing kits | B |
| LouisDitTrieau | 2009 | Reliability and patient acceptance for self-collected vaginal swabs for group B streptococcus screening in third trimester of pregnancy | B |
| Michel | 2009 | Pitfalls of internet-accessible diagnostic tests: inadequate performance of a CE-marked Chlamydia test for home use | I |
| Hicks | 2009 | Patient self-collection of group B streptococcal specimens during pregnancy | F |
| Brabin | 2009 | Delivery of chlamydia screening to young women requesting emergency hormonal contraception at pharmacies in Manchester, UK: a prospective study | A |
| Andersen | 2009 | Randomized Population-Based Study on Chlamydia Trachomatis Screening | B |
| Graseck | 2010 | Home compared with clinic-based screening for sexually transmitted infections: A randomized controlled trial | A |
| Hoyme | 2010 | Prevention of preterm birth is possible by vaginal pH screening, early diagnosis of bacterial vaginosis or abnormal vaginal flora and treatment | F |
| Lange | 2010 | Cervical cancer screening by self-sampling for p16 with two different sampling systems | B |
| Peipert | 2010 | Home screening compared with clinic-based screening for sexually transmitted infections | A |
| Siegmund-Schultze | 2010 | Prevention of preterm birth by pH-self assessment: A prospective controlled trial | B |
| Sutcliffe | 2010 | Prevalence and correlates of Trichomonas vaginalis infection among female US federal prison inmates | A |
| Falk | 2010 | Sampling for Chlamydia trachomatis infection - a comparison of vaginal, first-catch urine, combined vaginal and first-catch urine and endocervical sampling | A |
| Sacks-Davis | 2010 | Home-based chlamydia testing of young people attending a music festival--who will pee and post? | A |
| Sungkar | 2010 | Study of Antenatal Model to Prevent Preterm Delivery | B |
| Andersen | 2011 | Impact of intensified testing for urogenital Chlamydia trachomatis infections: a randomised study with 9-year follow-up | A |
| Baraitser | 2011 | 'Do it yourself' sexual health care: the user experience | B |
| Bitzer | 2011 | Self-testing of vaginal pH to prevent preterm delivery: a controlled trial | E |
| Huppert | 2011 | Acceptability of self-testing for trichomoniasis increases with experience | A |
| Kock | 2011 | Detection of trichomonas vaginalis in HIV positive women in Pretoria, South Africa | B |
| Mitchell | 2011 | Assessing women's willingness to collect their own cervical samples for HPV testing as part of the ASPIRE cervical cancer screening project in Uganda | A |
| Taylor | 2011 | Clinical evaluation of the BD ProbeTec TM Chlamydia trachomatis Qx amplified DNA assay on the BD Viper TM system with XTR TM technology | G |
| VanDerPol | 2011 | Vaginal swabs are the optimal sample for screening women for chlamydial and gonorrheal infection using the Roche Cobas 4800 system | A |
| Xu | 2011 | Use of home-obtained vaginal swabs to facilitate rescreening for Chlamydia trachomatis infections: Two randomized controlled trials | C |
| Gaydos | 2011 | Chlamydia trachomatis age-specific prevalence in women who used an internet-based self-screening program compared to women who were screened in family planning clinics | A |
| Gaydos | 2011 | Trichomonas vaginalis infection in women who submit self-obtained vaginal samples after internet recruitment | A |
| Trappe | 2011 | Vaginal-perianal compared with vaginal-rectal cultures for detecting group B streptococci during pregnancy | I |
| Bourgeois-Nicolaos | 2012 | Assessment of Neisseria gonorrhoeae Chlamydia trachomatis simultaneous detection by real-time PCR according to the care unit | B |
| Broberg | 2012 | Increasing participation in cervical cancer screening: Telephone call to long time abstaining women in Sweden. Results from RACOMIP, a randomized controlled trial | A |
| Donders | 2012 | Acceptance of self-testing for increased vaginal pH in different subsets of Ugandan women | D |
| Poli | 2012 | cAREHPVTM experience in four countries | B |
| Rickard | 2012 | Candida in pregnancy study (CIPS): Protocol and progress on a randomised trial of treatment of asymptomatic vaginal candidiasis in pregnant women to prevent preterm birth | B |
| Stanczuk | 2012 | Validation of Coba 4800 HPV detection in cervical and self-collected vaginal samples from women with abnormal cervical cytology in dumfries and galloway, Scotland | B |
| Roth | 2012 | A methodological approach to improve the sexual health of vulnerable female populations: incentivized peer-recruitment and field-based STD testing | A |
| Aziz | 2013 | The effectiveness and acceptability of self-sampling against conventional PAP smear in University Malaya Medical Centre (UMMC) | B |
| Cabeza | 2013 | Chlamydia trachomatis screening and treatment in pregnant women in Lima, Peru | B |
| Geraets | 2013 | Clinical evaluation of high-risk HPV detection on self-samples using the indicating FTA-elute solid-carrier cartridge | A |
| Hocking | 2013 | Advances in sampling and screening for chlamydia | B |
| Jentschke | 2013 | Enzyme-linked immunosorbent assay for p16INK4a - A new triage test for the detection of cervical intraepithelial neoplasia? | A |
| Jentschke | 2013 | Evaluation of a multiplex real time PCR assay for the detection of human papillomavirus infections on self-collected cervicovaginal lavage samples | A |
| Law | 2013 | A comparative study assessing the efficacy and acceptability of anorectal swabs for antenatal GBS screening | A |
| Madhivanan | 2013 | Feasibility and acceptability of self-collected vaginal swabs for diagnosis of bacterial vaginosis among pregnant women in a community setting in Rural Mysore, India | B |
| Ogilvie | 2013 | Results of a community-based cervical cancer screening pilot project using human papillomavirus self-sampling in Kampala, Uganda | A |
| Ostensson | 2013 | Projected cost-effectiveness of repeat high-risk human papillomavirus testing using self-collected vaginal samples in the Swedish cervical cancer screening program | A |
| Sancho-Garnier | 2013 | HPV self-sampling or the Pap-smear: a randomized study among cervical screening nonattenders from lower socioeconomic groups in France | A |
| Stewart | 2013 | Comparing swabs for diagnosing chlamydia and gonorrhea in women: Recent findings | B |
| Sungkar | 2013 | Early self-diagnosis and treatment of bacterial vaginosis to prevent preterm premature rupture of membranes | B |
| Ting | 2013 | High-risk human papillomavirus messenger RNA testing in physician- And self-collected specimens for cervical lesion detection in high-risk women, Kenya | A |
| Faro | 2013 | Accuracy of an accelerated, culture-based assay for detection of group B streptococcus | J |
| Lawton | 2013 | Nucleic acid amplification tests of self-taken vulvovaginal swabs are more sensitive than clinician taken endocervical culture for gonorrhoea | A |
| Loaring | 2013 | Could a peer-led intervention increase uptake of chlamydia screening? A proof of principle pilot study | A |
| Verhoef | 2013 | A second generation cervico-vaginal lavage device shows similar performance as its preceding version with respect to DNA yield and HPV DNA results | A |
| Gertig | 2013 | Home-based human papillomavirus (HPV) self-sampling for improving participation in cervical screening | C |
| Castle | 2014 | Clinical determinants of a positive visual inspection after treatment with acetic acid for cervical cancer screening | C |
| Ducancelle | 2014 | Interest of human papillomavirus DNA quantification and genotyping in paired cervical and urine samples to detect cervical lesions | A |
| Franciscatto | 2014 | Comparison of urine and self-collected vaginal samples for detecting human papillomavirus DNA in pregnant women | E |
| Kamal | 2014 | HPV detection in a self-collected vaginal swab combined with VIA for cervical cancer screening with correlation to histologically confirmed CIN | C |
| Keegan | 2014 | Chlamydia trachomatis Infection: Screening and Management | B |
| Li | 2014 | Comparison of flocked and Aptima swabs and two specimen transport media in the Aptima combo 2 assay | A |
| Mandigo | 2014 | Community health workers paired with human papillomavirus self-samplers a promising method to reduce cervical cancer | B |
| Mantzana | 2014 | Applicability of self-obtained urine and vaginal samples for HPV-17, -18, -31 and -45 cervical cancer screening in pregnancy: A pilot cross-sectional study | F |
| Mount | 2014 | Self-administered GBS testing in pregnant women | B |
| Nelson | 2014 | Screening for Gonorrhea and Chlamydia: Systematic Review to Update the U.S. Preventive Services Task Force Recommendations [Internet] | B |
| Peedicayil | 2014 | The community prevalence of human papillomavirus (HPV) in India and the feasibility of self-collected vaginal swabs | B |
| Rosenbaum | 2014 | Acceptability of self-collected versus provider-collected sampling for HPV DNA testing among women in rural El Salvador | C |
| Verhoef | 2014 | Triage by methylation-marker testing versus cytology in women who test HPV-positive on self-collected cervicovaginal specimens (PROHTECT-3): A randomised controlled non-inferiority trial | A |
| Virtanen | 2014 | Self-sampling experiences among non-attendees to cervical screening | D |
| Haguenoer | 2014 | Vaginal self-sampling is a cost-effective way to increase participation in a cervical cancer screening programme: a randomised trial | A |
| Bretelle | 2014 | Medico-economic Impact of Screening Atopobium Vaginae and Gardnerella Vaginalis in Molecular Biology by "Point-of-care" During Pregnancy | B |
| Badman | 2015 | Point-of-care testing and immediate treatment of curable sexually transmitted and genital infections among antenatal women in Papua New Guinea | B |
| Bhatla | 2015 | Feasibility and reliability of cervical cancer screening by HPV DNA testing of self-collected samples in a north Indian community | B |
| Bosgraaf | 2015 | Comparative performance of novel self-sampling methods in detecting high-risk human papillomavirus in 30,130 women not attending cervical screening | C |
| Cabeza | 2015 | Feasibility of Chlamydia trachomatis screening and treatment in pregnant women in Lima, Peru: a prospective study in two large urban hospitals | D |
| Chang | 2015 | Concordance analysis of methylation biomarkers between self-collected and physician-collected samples in cervical neoplasm | C |
| Ducancelle | 2015 | Home-based urinary HPV DNA testing in women who do not attend cervical cancer screening clinics | A |
| Hebl | 2015 | Perceptions and acceptability of self-testing for human papillomavirus among women presenting for cervical cancer screening | B |
| Lorenzi | 2015 | Effectiveness of cervicovaginal self-collection in the screening of cervical cancer | B |
| Masese | 2015 | Screening for sexually transmitted infections in adolescent girls and young women in Mombasa, Kenya | B |
| Moinon | 2015 | Feasibility of self-collected sampling for human papillomavirus (HPV) DNA testing among women in a rural area | B |
| Moses | 2015 | Towards the best model for cervical cancer screening in low and middle income countries: Results from a pilot randomized controlled trial comparing self-collected HPV testing with VIA in Uganda | B |
| Oranratanaphan | 2015 | Acceptability of self-sampling HPV testing among women for cervical cancer screening | B |
| Priddle | 2015 | Results of a 2014 QLD trial of pharmacy accessed testing for chlamydia trachomatis via retail self-test URI swab postal specimen kits | B |
| Virtanen | 2015 | The costs of offering HPV-testing on self-taken samples to non-attendees of cervical screening in Finland | A |
| Witkin | 2015 | The vaginal microbiome, vaginal anti-microbial defence mechanisms and the clinical challenge of reducing infection-related preterm birth | B |
| Cadman | 2015 | Attitudes towards cytology and human papillomavirus self-sample collection for cervical screening among Hindu women in London, UK: a mixed methods study | A |
| Moses | 2015 | Uptake of community-based, self-collected HPV testing vs. visual inspection with acetic acid for cervical cancer screening in Kampala, Uganda: preliminary results of a randomised controlled trial | A |
| Tingey | 2015 | Self-administered sample collection for screening of sexually transmitted infection among reservation-based American Indian youth | A |
| Donders | 2016 | Screening for abnormal vaginal microflora by self-assessed vaginal pH does not enable detection of sexually transmitted infections in Ugandan women | G |
| Donders | 2016 | Increased vaginal pH in Ugandan women: what does it indicate? | G |
| Enerly | 2016 | Self-sampling for human papillomavirus testing among non-attenders increases attendance to the norwegian cervical cancer screening programme | A |
| Ko | 2016 | A questionnaire study on the acceptability of self-sampling versus screening by clinicians for Group B Streptococcus | D |
| Krings | 2016 | Comparison of delphi screener and evalyn brush self-samplers for HPV screening in low resource settings (accessing*) | B |
| Lee | 2016 | Did self-sampling improve the adherence to group B streptococci screening in pregnant women? | B |
| Smartlowit-Briggs | 2016 | Community-Based Assessment to Inform a Chlamydia Screening Program for Women in a Rural American Indian Community | D |
| Wynn | 2016 | Acceptability and Feasibility of Sexually Transmitted Infection Testing and Treatment among Pregnant Women in Gaborone, Botswana, 2015 | D |
| vanRooijen | 2016 | Young Low-Risk Heterosexual Clients Prefer a Chlamydia Home Collection Test to a Sexually Transmitted Infection Clinic Visit in Amsterdam, the Netherlands, A Cross-Sectional Study | A |
| Vassilakos | 2016 | Use of swabs for dry collection of self-samples to detect human papillomavirus among Malagasy women | A |
| Vallely | 2016 | Point-of-care testing and treatment of sexually transmitted infections to improve birth outcomes in high-burden, low-income settings | H |
| Viviano | 2016 | A Comparison of Cotton and Flocked Swabs for Vaginal Self Collection | B |
| Asciutto | 2017 | Vaginal and Urine Self-sampling Compared to Cervical Sampling for HPV-testing with the Cobas 4800 HPV Test | A |
| Bristow | 2017 | Chlamydia trachomatis, Neisseria gonorrhoeae, and Trichomonas vaginalis screening and treatment of pregnant women in Port-au-Prince, Haiti | D |
| Burger | 2017 | The cost-effectiveness of cervical self-sampling to improve routine cervical cancer screening: The importance of respondent screening history and compliance | A |
| Cremer | 2017 | Scale-Up of an Human Papillomavirus Testing Implementation Program in El Salvador | C |
| Donders | 2017 | Role of Molecular Biology in Diagnosis and Characterization of Vulvo-Vaginitis in Clinical Practice | B |
| Lagier | 2017 | Vaginal self-sampling as a diagnosis tool in low-income countries and potential applications for exploring the infectious causes of miscarriage | B |
| Lee | 2017 | The impact of screening and treatment of maternal genitourinary tract infections on preterm birth and small for gestational age in rural Bangladesh | B |
| Mbatha | 2017 | Self-sampling for human papillomavirus testing among rural young women of KwaZulu-Natal, South Africa | C |
| Morikawa | 2017 | Acceptability of antenatal sexually transmitted infection screening in South African human immunodeficiency virusinfected pregnant women | B |
| Mudau | 2017 | High prevalence of asymptomatic sexually transmitted infections among HIV-infected pregnant women in South Africa | B |
| Nguyen | 2017 | Prevalence of sexually transmitted infections and acceptability, feasibility of screening in antenatal care, Vietnam, 2016-2017 | B |
| Wiesenfeld | 2017 | Screening for Chlamydia trachomatis Infections in Women | B |
| McDowell | 2017 | Cervical Cancer Screening Preferences Among Trans-Masculine Individuals: Patient-Collected Human Papillomavirus Vaginal Swabs Versus Provider-Administered Pap Tests | A |
| Reisner | 2017 | Comparing self- and provider-collected swabbing for HPV DNA testing in female-to-male transgender adult patients: a mixed-methods biobehavioral study protocol | A |
| Wilson | 2017 | Internet-accessed sexually transmitted infection (e-STI) testing and results service: A randomised, single-blind, controlled trial | A |
| Wiesenfeld | 2017 | STD Testing in Outpatient Practices | C |
| Abdullah | 2018 | Human Papilloma Virus (HPV) self-sampling: do women accept it? | C |
| Francis | 2018 | Prevalence of sexually transmitted infections among young people in South Africa: A nested survey in a health and demographic surveillance site | A |
| Ivanus | 2018 | Randomised trial of HPV self-sampling among non-attenders in the Slovenian cervical screening programme ZORA: Comparing three different screening approaches | C |
| Leinonen | 2018 | Safety and acceptability of human papillomavirus testing of self-collected specimens: A methodologic study of the impact of collection devices and HPV assays on sensitivity for cervical cancer and high-grade lesions | A |
| Morikawa | 2018 | Acceptability and Feasibility of Integrating Point-of-Care Diagnostic Testing of Sexually Transmitted Infections into a South African Antenatal Care Program for HIV-Infected Pregnant Women | D |
| Nassie | 2018 | Group B Strep screening-who is more accurate: The patient or the physician? | B |
| Pearson | 2018 | Perceptions of and willingness to use chlamydia and gonorrhea self-testing kits | B |
| Pearson | 2018 | Improving STD service delivery: Would American patients and providers use self-tests for gonorrhea and chlamydia? | A |
| Reisner | 2018 | Test performance and acceptability of self-versus provider-collected swabs for high-risk HPV DNA testing in female-to-male trans masculine patients | A |
| Senkomago | 2018 | High-risk HPV-RNA screening of physician- and self-collected specimens for detection of cervical lesions among female sex workers in Nairobi, Kenya | A |
| Zhang | 2018 | Durability of clinical performance afforded by self-collected HPV testing: A 15-year cohort study in China | C |
| Peterman | 2018 | Preparing for the Chlamydia and Gonorrhea Self-Test | B |
| Huchko | 2018 | Cervical cancer screening through human papillomavirus testing in community health campaigns versus health facilities in rural western Kenya | A |
| Adsul | 2019 | A community-based, cross-sectional study of hrHPV DNA self-sampling-based cervical cancer screening in rural Karnataka, India | C |
| Brewer | 2019 | Acceptability of human papillomavirus self-sampling for cervical-cancer screening in under-screened maori and pasifika women: A pilot study | A |
| Furfaro | 2019 | Detection of group B Streptococcus during antenatal screening in Western Australia: a comparison of culture and molecular methods | E |
| Gizaw | 2019 | Uptake of Cervical Cancer Screening in Ethiopia by Self-Sampling HPV DNA Compared to Visual Inspection with Acetic Acid: A Cluster Randomized Trial | C |
| Nguyen | 2019 | Acceptability and feasibility of sexually transmissible infection screening among pregnant women in Hanoi, Vietnam | D |
| Rocha | 2019 | T. vaginalis in riverside women in Amazonia, Brazil: An experience using the EVALYN BRUSH vaginal self-collection device | C |
| Basu | 2019 | A pilot study to evaluate home-based screening for the common non-communicable diseases by a dedicated cadre of community health workers in a rural setting in India | C |
| Lorenzi | 2019 | Age-related acceptability of vaginal self-sampling in cervical cancer screening at two university hospitals: a pilot cross-sectional study | C |
| McLarty | 2019 | Cervical Human Papillomavirus Testing With Two Home Self-Collection Methods Compared With a Standard Clinically Collected Sampling Method | C |
| Jensen | 2019 | Comparing Specimen Collection Techniques to Screen for Sexually Transmitted Infections in Pregnant Women: A Pilot Study | K |
| Adcock | 2019 | Acceptability of self-taken vaginal HPV sample for cervical screening among an under-screened Indigenous population | A |
| Bakiewicz | 2020 | "the best thing is that you are doing it for yourself" - Perspectives on acceptability and feasibility of HPV self-sampling among cervical cancer screening clients in Tanzania: A qualitative pilot study | C |
| Baxter | 2020 | Feasibility of patient-collected tampon samples for longitudinal monitoring of the vaginal microbiome | B |
| Haque | 2020 | Eliminating cervical cancer in Mali and Senegal, two sub-Saharan countries: Insights and optimizing solutions | B |
| Hoyme | 2020 | Reduced incidence of early preterm birth in the State of Thuringia following an intravaginal pH-self-monitoring screening program | B |
| Islam | 2020 | High-risk Human Papillomavirus Messenger RNA Testing in Wet and Dry Self-collected Specimens for High-grade Cervical Lesion Detection in Mombasa, Kenya | C |
| Kuriakose | 2020 | Diagnostic accuracy of self-collected vaginal samples for HPV DNA detection in women from South India | A |
| Lefeuvre | 2020 | Urinary HPV DNA testing as a tool for cervical cancer screening in women who are reluctant to have a Pap smear in France | A |
| Poli | 2020 | Feasibility, Acceptability, and Efficacy of a Community Health Worker-Driven Approach to Screen Hard-to-Reach Periurban Women Using Self-Sampled HPV Detection Test in India | C |
| Rohner | 2020 | Racial and Ethnic Differences in Acceptability of Urine and Cervico-Vaginal Sample Self-Collection for HPV-Based Cervical Cancer Screening | C |
| VanDerPol | 2020 | Evaluation of the Performance of a Point-of-Care Test for Chlamydia and Gonorrhea | A |
| Desai | 2020 | Design and feasibility of a novel program of cervical screening in Nigeria: self-sampled HPV testing paired with visual triage | C |
| Goldstein | 2020 | A rapid, high-volume cervical screening project using self-sampling and isothermal PCR HPV testing | C |
| Goldstein | 2020 | Patient Satisfaction With Human Papillomavirus Self-Sampling in a Cohort of Ethnically Diverse and Rural Women in Yunnan Province, China | A |
| Goldstein | 2020 | Improved Rates of Cervical Cancer Screening Among Transmasculine Patients Through Self-Collected Swabs for High-Risk Human Papillomavirus DNA Testing | A |
| Hawkes | 2020 | Self-Collection for Cervical Screening Programs: From Research to Reality | B |
| Maza | 2020 | Cervical Cancer Screening with Human Papillomavirus Self-Sampling Among Transgender Men in El Salvador | A |
| ArandaFlores | 2021 | Self-collected versus clinician-collected cervical samples for the detection of HPV infections by 14-type DNA and 7-type mRNA tests | C |
| Cantor | 2021 | Screening for Chlamydial and Gonococcal Infections: A Systematic Review Update for the U.S. Preventive Services Task Force | B |
| Cantor | 2021 | Screening for Chlamydial and Gonococcal Infections: Updated Evidence Report and Systematic Review for the US Preventive Services Task Force | B |
| Galvez | 2021 | Concordance between self-sampling and standar endocervical sample collection to identify sexual transmission infections in an urban-rural area of peru | A |
| Guo | 2021 | [Performance of vaginal self-sampling high-risk HPV genotyping as primary and combining cytology or viral load as secondary in cervical cancer screening] | C |
| Hellsten | 2021 | Equal prevalence of severe cervical dysplasia by HPV self-sampling and by midwife-collected samples for primary HPV screening: a randomised controlled trial | A |
| Klischke | 2021 | Performance of a six-methylation-marker assay on self-collected cervical samples - A feasibility study | C |
| Lozar | 2021 | Cervical cancer screening postpandemic: Self-sampling opportunities to accelerate the elimination of cervical cancer | B |
| Mabonga | 2021 | Prevalence and predictors of asymptomatic Chlamydia trachomatis and Neisseria gonorrhoeae in a Ugandan population most at risk of HIV transmission | D |
| Naicker | 2021 | Strong correlation between urine and vaginal swab samples for bacterial vaginosis | E |
| Ornskov | 2021 | Clinical performance and acceptability of self-collected vaginal and urine samples compared with clinician-taken cervical samples for HPV testing among women referred for colposcopy. A cross-sectional study | A |
| Shen | 2021 | Clinical Evaluation of a Self-Testing Kit for Vaginal Infection Diagnosis | G |
| Singh | 2021 | Barriers to compliance for cervical screening among HPV vaccinated women in low-resource settings | B |
| Yahaya | 2021 | "No one left behind"-Incorporating HPV screening test into Orang Asli outreach program | C |
| Pritt | 2021 | Home Self-collection and Specimen Pooling: Tools for Convenient and Economical Detection of Sexually Transmitted Infections | B |
| Choi | 2022 | Uptake and correlates of cervical cancer screening among women attending a community-based multi-disease health campaign in Kenya | D |
| Dawson | 2022 | Self-care interventions for sexual and reproductive health in humanitarian and fragile settings: a scoping review | B |
| Eche | 2022 | Knowledge, attitude and practice of female university students regarding human papillomavirus and self-sampling in KwaZulu-Natal, South Africa: a cross-sectional survey | A |
| Hamers | 2022 | Updated evidence-based recommendations for cervical cancer screening in France | B |
| Zhao | 2022 | [Performance of point-of-care testing for cervical cancer screening] | C |
| Avian | 2022 | Clinical validation of full HR-HPV genotyping HPV Selfy assay according to the international guidelines for HPV test requirements for cervical cancer screening on clinician-collected and self-collected samples | C |
| Dawkins | 2022 | Clinical Integration of a Highly Accurate Polymerase Chain Reaction Point-of-Care Test Can Inform Immediate Treatment Decisions for Chlamydia, Gonorrhea, and Trichomonas | A |
| Horberg | 2022 | Patient Demographics and the Utilization of a Novel 3-Anatomic-Site Testing Panel Including Rectal Self-Collection as Compared With Usual Care Testing | A |
| Mamo | 2022 | Human papillomavirus self-sampling: A tool in cancer prevention and sexual health promotion | B |
| Rosenfeld | 2022 | In diagnosing BV, are patient-collected samples as accurate as provider-collected samples? | B |
| Ibáñez | 2022 | Acceptability of Self-sampling for Cervical Cancer Screening | C |

| **Code** | **Reason for exclusion (n = number of studies)** |
| --- | --- |
| A | Not stated or unclear if population includes pregnant women (n = 72) |
| B | Wrong study design (n = 69) |
| C | Population did not include pregnant women (n = 37) |
| D | Single arm design (n = 14) |
| E | Wrong comparator (n = 9) |
| F | Self-collected and provider-collected samples taken from different non-randomised participants (n = 9) |
| G | Pregnant women not reported separately (n = 6) |
| H | Ongoing study (n = 2) |
| I | Wrong intervention (n = 2) |
| J | Wrong outcome(s) (n = 2) |
| K | Study terminated (n = 1) |
