# Supplementary material for: Self-collection of samples for group B streptococcus testing during pregnancy: a systematic review and meta-analysis
Source: BMC Med. 2023 Dec 18;21:498. doi: 10.1186/s12916-023-03186-x (PMC10729404; doi:10.1186/s12916-023-03186-x)
Supplement: Supplementary file 5 — Additional file 5: Table S1. Research/trial integrity assessment [file 12916_2023_3186_MOESM5_ESM.docx]

## Additional file 5: Research integrity assessment

Research integrity assessments of included randomised trials (n=4) are summarised in Table S1. There were no expressed concerns or retractions for any included studies (Domain 1). For Domain 2: trial registration, only one trial (30) provided a trial registry number but was retrospectively registered. The other trials did not provide trial registry numbers, however, two of these studies (19, 38) were commenced prior to 2005 when the WHO International Clinical Trials Registry Platform was established. For Domain 3: ethics approval, one trial (38) did not provide an ethics approval number, and no author contact details were available to request this information. For Domain 4: author group, one trial (38) only had two authors which was not deemed a plausible number for the study design. For Domain 5: methods, two studies (30 , 38) did not sufficiently describe the randomisation process, with one of these studies (38) not detailing the number of participants in each group, nor providing baseline details for each group to allow assessment of whether randomisation worked and whether there was excessive similarity or difference in the characteristics of the study participants between groups (Domain 5 and Domain 6: results). Three (30, 35, 38) out of four trials were given an overall RIA decision of “awaiting classification – some concern”, with concerns regarding integrity in one or more domain(s).

**Table S1: Trial integrity assessment**

|  | **Domain 1: Retraction or expression of concern** | **Domain 2: Trial registration** | **Domain 3: Ethics approval** | **Domain 4: Author group** | **Domain 5: Methods** | **Domain 6: Results** | **Decision (Overall RIA)** |
| --- | --- | --- | --- | --- | --- | --- | --- |
| **Camus 2021 (30)** | No concern | Exclude | No concern | No concern | Exclude | No concern | **Some concern** |
| **Price 2006 (19)** | No concern | N/A* | No concern | No concern | No concern | No concern | **No concern** |
| **Seto 2019 (35)** | No concern | Exclude | No concern | No concern | No concern | No concern | **Some concern** |
| **Torok 2000 (38)** | No concern | N/A* | Exclude | Exclude | Exclude | Exclude | **Some concern** |

**Trial commenced prior to 2005 when the WHO International Clinical Trials Registry Platform (ICTRP) was established*
