# Supplementary material for: Self-collection of samples for group B streptococcus testing during pregnancy: a systematic review and meta-analysis
Source: BMC Med. 2023 Dec 18;21:498. doi: 10.1186/s12916-023-03186-x (PMC10729404; doi:10.1186/s12916-023-03186-x)
Supplement: Supplementary file 6 — Additional file 6: Figure S1. Risk of bias assessment and applicability of the included studies. [file 12916_2023_3186_MOESM6_ESM.docx]

## Additional file 6: Risk of bias assessment and applicability of the included studies

Risk of bias regarding patient selection was high in one study (38), unclear in eight (17, 20, 30-32, 34-37) and low in two (19, 33). Unclear studies (17, 20, 30-32, 34-37) lacked sufficient participant recruitment detail like whether a consecutive or random selection of participants were enrolled, or the timeframe in which enrolment occurred. The one study deemed at high risk enrolled participants in an alternating fashion (38).

Risk of bias regarding reporting and execution of tests (description of cut-off or blinding of the self-collected sample test [index test] towards the provider-collected sample test [reference test]) was high in five studies (20, 30, 32-34, 37), unclear in one (19), and low in five (17, 31, 35, 36, 38). Three studies (30, 32, 33) did not provide a threshold for test interpretation. Risk of bias regarding verification with testing of a provider-collected sample (acceptable validity or blinding towards tests) was high in two studies (20, 34, 37), unclear in two (19, 30), and low in seven (17, 31-33, 35, 36, 38). Two studies (20, 34, 37) did not blind lab staff to sample collection method and two studies (19, 30) did not mention whether blinding occurred.

Risk of bias regarding flow and timing of self-collection and provider-collection was high in four studies (31, 33-35) and low in seven (17, 19, 20, 30, 32, 36-38). The delay between self-sampling and verification with the reference standard, provider sampling, was short, with all self-collected and provider-collected swabs taken on the same day, except for one study (31), where samples may have been taken at different visits. Three studies (33-35) did not include all participants in their analyses; withdrawal of participants was not explained in two of these studies (34, 35), and the other study (33) adequately detailed the reasons, but did not adjust for potential resulting bias.

**Figure S1: Risk of bias assessment and applicability of the included studies**

**
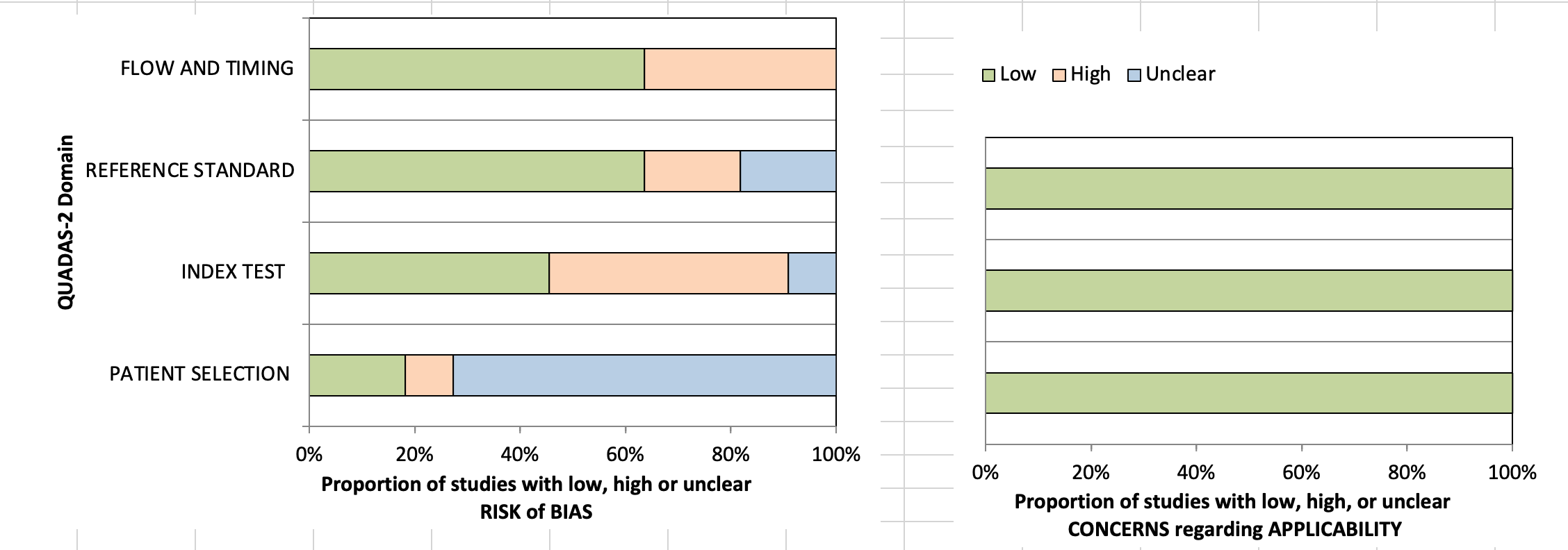

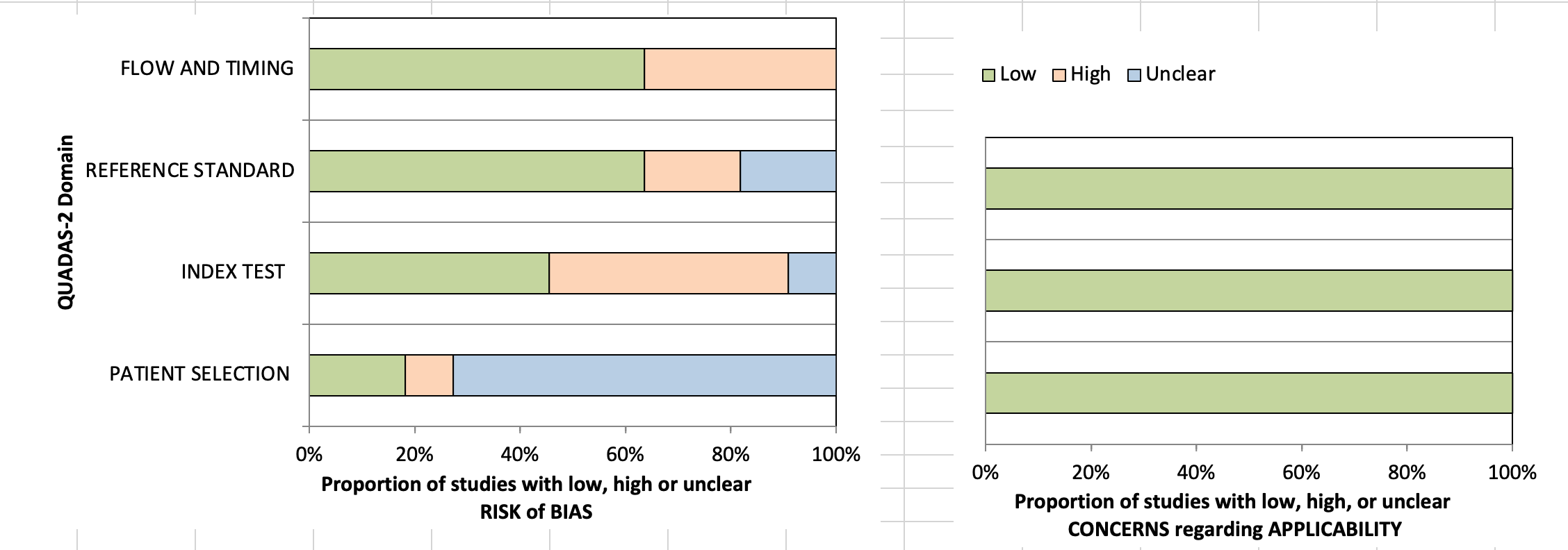
**
