# Supplementary material for: Self-collection of samples for group B streptococcus testing during pregnancy: a systematic review and meta-analysis
Source: BMC Med. 2023 Dec 18;21:498. doi: 10.1186/s12916-023-03186-x (PMC10729404; doi:10.1186/s12916-023-03186-x)
Supplement: Supplementary file 7 — Additional file 7. Sensitivity analyses. [file 12916_2023_3186_MOESM7_ESM.docx]

## Additional file 7: Sensitivity analyses

A sensitivity analysis of non-randomised studies, in which we excluded studies that did not incubate all samples in enriched culture media (33), did not change the results, except that heterogeneity for sensitivity was lower (participants = 1,854; studies = 6; Se 0.90, 95% CI: 0.83-0.94, Sp 0.97, 95% CI: 0.93-0.99; Se I^2^ = 24.84%, Sp I^2^ = 75.30%).

A sensitivity analysis of non-randomised studies, that excluded studies with participants at gestational ages less than 35 weeks (20, 32, 36, 37) did not change the results (participants = 1,389; studies = 4; Se 0.94, 95% CI: 0.80-0.98, Sp 0.98, 95% CI: 0.93-0.99; Se I^2^ = 35.86%, Sp I^2^ = 68.68%).
