# Supplementary material for: Self-collection of samples for group B streptococcus testing during pregnancy: a systematic review and meta-analysis
Source: BMC Med. 2023 Dec 18;21:498. doi: 10.1186/s12916-023-03186-x (PMC10729404; doi:10.1186/s12916-023-03186-x)
Supplement: Supplementary file 8 — Additional file 8: Table S2. Summary of additional outcomes. [file 12916_2023_3186_MOESM8_ESM.docx]

## Additional file 8: Summary of additional outcomes

Uptake of self-collection was reported in two studies, in which 9.0% (n=21/234) (33) and 14.5% (n= 56/386) (19) of women invited to participate in the studies declined. Reasons cited were that they: preferred sampling to be performed by the healthcare provider (54.8% (n=17/31) (19); 14.3% (n=3/21) (33)); thought self-collection would be uncomfortable (23.8% (n=5/21) (33)); felt uncomfortable with self-collection (68.2% (n=15/22) (19)); or reported feeling physically unable to (16.1% (n=5/31) (19)). Nebreda-Martin et al (33) assessed participants who undertook self-collection at home; participants were given the materials for sample collection and a satisfaction survey at their 30-32 week consultation, three to seven weeks prior to their 35-37 week consultation, and instructed to collect their self-collect their sample the same morning of their appointment. Of women who agreed to participate in the study, 3.3% (n=7/213) forgot to bring either their self-collected sample or the accompanying survey to the appointment.

Of the five studies (19, 30, 35, 36, 38) with cross-over designs that determined order of self-collection and provider-collection, four (30, 35, 36, 38) reported outcomes of sampling order. No statistically significant difference in test accuracy was observed between the two groups with different orders of sampling sequences in any of the studies.

Five studies (17, 31, 34, 35, 38) reported on proportion of infections treated, and in four studies (17, 31, 35, 38) 100% of participants with positive tests were given antibiotics. In one study (34) a proportion of participants with positive test results and a proportion with negative results were given antibiotics, but these data were not disaggregated by whether these test results were from self-collected or provider-collected samples.

Three studies (19, 31, 33) reported on acceptability of self-sampling, with the majority of participants reporting feeling comfortable with self-collection (84.2%, n=160/190 (33)) or rating self-collection as acceptable (81.5%, n=251/308 (19)), and less participants reporting pain with self-collection compared to provider-collection (19.6% v 11.9% (31)).

Eight studies reported on preference for self-sampling. In four studies (17, 19, 31, 35), participants preferred provider-collection (43.20% v 28.5% (17); 25.3% v 20.9% (31); 43.3% v 13.0% (35); 27.25% v 22.75% (19)), and in the other four studies (20, 30, 32, 37, 38), participants preferred self-collection (46.9% v 17.4% (30); 57.7% v 37% (20, 37); 34% v 26% (32); 58.0% (38)).

Six studies (19, 20, 30, 32, 33, 35, 37) reported on self-sampling feasibility. In Mercer & Taylor (20, 37), 90.4% (n=227/251) had no difficulty collecting self-samples. 9.6% (n=24/251) of participants found the self-collection technique difficult, of whom 62.5% (n=15/24) blamed difficulty on the size of the gravid uterus, 37.5% (n=9/24) stated that the ano-rectal site was difficult to reach for culture collection, and 4.2% (n=1/24) was afraid to insert the swab. Of 56.6% (n=142/251) who preferred self-collection, 6% reported that they are easy or easier to perform. Of the 37.1% (n=93/251) who preferred provider-collection, reasons included, they had doubts about their own ability to accurately perform the test (22.6%, n=21/93), culture sites were hard to reach (5.4%, n=5/93), and for 1.1% (n=1/93) labour interfered with self-collection. In Price (19), 14.2% (n=47/330) of participants were concerned about not doing self-collection correctly. In Nebreda-Martin (33) 72.6% (n=138/190) reported experiencing no difficulties and 27.3% (n=52/190) of participants reported encountering difficulty with self-collection, with some of the cited reasons being: not knowing if they are doing it correctly (16.8% n=32/190), difficulty inserting the swab into both holes (11%, n=21/190), difficulty due to the presence of haemorrhoids (3.1%, n=6/190), not easily locating the holes (1.6%, n=3/190), and difficulty handling the sampling instrument (1.1%, n=2/190). Seto (35) asked participants to rate themselves with a confidence score for self-collection; the mean score was 6.08/10 (± 2.4). 66.6% (n=273/410) of participants had perceived difficulty with self-collection. Listed reasons for having difficulty with self-collection included: difficulty placing the swab into the anus (81.6%, n=226/277) or vagina (15.9% (n=44/277), difficulty identifying the vagina and anus (5.4%, n=15/277), difficulty placing the samples in the container (2.9%, n=8/277), difficulty labelling the samples (0.7%, n=2/277), fear of procedure leading to difficulty (6.9%, n=19/277), and fear of pain leading to difficulty (21.7%, n=60/277). Participants had been provided with written information sheets and a video on self-collection; 88.4% (n=358/405) of participants reported that they found the written information sheet useful, and 96.1% (n=393/409) found the video helpful. More convenience was listed as a reason for preferring self-collection (56.8%, n=130/229) and as one of the reasons for preferring provider-collection 13.2% (n=47/355), and inconvenience was listed as one of the reasons for not preferring provider-collection (43.4%, n=23/53). Of those who did not prefer self-collection, reasons included: inconvenience n=40/187 (22.5%) and procedure difficulty n=36/187 (19.3%). In Camus (30), participants cited "easy to use" (98.1%, n=105/107) and "cheaper alternative" (4.7%, n=5/106) as reasons to prefer self-collection, and "hard to process self-sampling" (15.4%, n=6/39) and "medical assistance" (84.6%, n=33/39) as reasons to prefer provider-collection. A survey that included 161 participants in Molnar (32), found that of 25.5% (n=41/161) who preferred provider-collection, n=8/13 of these participants expressed concern that they would not obtain as accurate a result as their healthcare provider.

A summary of these outcomes is displayed in Table S2.

**Table S2: Summary of additional outcomes**

| **Author, Year** | **Uptake of self-collection** | | | **Sampling order** | **Linkage to treatment** | **Acceptability** | **Preference** | | | | | | | **Feasibility** | |
| --- | --- | --- | --- | --- | --- | --- | --- | --- | --- | --- | --- | --- | --- | --- | --- |
|  | **% of participants who declined self-collection** | **Reasons for declining self-collection** | **Proportion who brought self-sample for testing if taken outside of the clinic** | **Statistically significant difference in test accuracy between groups with different sampling order sequences?** | **Proportion of participants given antibiotics** |  | **% of participants who preferred self-collection** | **Reasons for preferring self-collection** | **% of participants who preferred provider-collection** | **Reasons for preferring provider-collection** | **% of participants who would recommend self-collection** | **% of participants who would not recommend self-collection** | **% of participants who would recommend provider-collection** | **Ease of self-collection** | **Difficulty with self-collection** |
| **Arya 2008 (17)** | Not measured | Not measured | N/A | N/A | 100% | Not measured | 28.5% | Not measured | 43.2% | Not measured | 26.5% | Not measured | 45.2% | Not measured | Not measured |
| **Camus 2021 (30)** | Not measured | Not measured | N/A | No | Not measured | Not measured | 46.9% | Of those who preferred self-collection (46.9%, n=105/224), 12.3% (n=13/106) selected "in accordance with religious and cultural norms" as a reason  Of those who preferred provider-collection (17.4%, 39/224), 30.8% (n=12/39) selected "physical discomfort", and 30.8% (n=12/39) selected "embarrassment" as reasons | 17.4% | Not measured | 88.8% | 11.2% | Not measured | Participants cited "easy to use" (98.1%, n=105/107) and "cheaper alternative" (4.7%, n=5/106) as reasons to prefer self-collection | Participants cited "hard to process self-sampling" (15.4%, n=6/39) and "medical assistance" (84.6%, n=33/39) as reasons to prefer provider-collection |
| **Chen 2021 (31)** | Not measured | Not measured | N/A | N/A | 100% | 11.9% (n=62/521) reported more pain during self-sampling,  68.5% (n=357/521) reported equal level pain,  19.6% (n=102/521) reported more pain with provider-collection | 20.9% | Not measured | 25.3% | Not measured | Not measured | Not measured | Not measured | Not measured | Not measured |
|  |  |  |  |  |  |  |  |  |  |  |  |  |  |  |  |
| **Mercer 1995 (20) & Taylor 1997 (37)** | Not measured | Not measured | N/A | N/A | Not measured | Not measured | 57.7% | Of 56.6% (n=142/251) who preferred self-collection, 57.0% (n=81/142) reported this was due to privacy, 9.9% (n=14/142) due to greater physical comfort, and 4.2% (n = 6/142) due to both physical comfort and privacy | 37.0% | Of 37.1% (n=93/251) who preferred provider-collection, 10.8% (n=10/93) reported this was due to greater physical comfort and less discomfort | 90% expressed a desire to be offered self-sampling in the future; 79% stated their desire to have a choice about self-culture in the future; 89% believed that others would also like this choice | Not measured | Of those who preferred provider-collection, 65% still wanted the choice of collection method in the future. | 90.4% (n=227/251) had no difficulty collecting self-samples  Of 56.6% (n=142/251) who preferred self-collection, 6% reported that it is easy or easier to perform as a reason | 9.6% (n=24/251) of participants found the self-collection technique difficult, of whom 62.5% (n=15/24) blamed difficulty on the size of the gravid uterus, 37.5% (n=9/24) stated that the ano-rectal site was difficult to reach for culture collection, and 4.2% (n=1/24) were afraid to insert the swab Of the 37.1% (n=93/251) who preferred provider-collection, reported reasons included, they had doubts about their own ability to accurately perform self-collection (22.6%, n=21/93), culture sites were hard to reach (5.4%, n=5/93), and for 1.1% (n=1/93) labour interfered with self-collection |
| **Molnar 1997 (32)** | Not measured | Not measured | N/A | N/A | Not measured | Not measured | 34.0% | Not measured | 26.0% | Not measured | Not measured | Not measured | Not measured | Not measured | Of 25.5% (n=41/161) who preferred provider-collection, n=8/13 of these participants expressed concern that they would not obtain as accurate a result as their healthcare provider |
| **Nebreda-Martin 2022 (33)** | 9.0% (n=21/234) | 14.3% (n=3/21) preferred sampling to be performed by the healthcare provider; 23.8% (n=5/21) thought self-collection would be uncomfortable | 3.3% (n=7/213) forgot to bring their self-collected sample or the accompanying survey to the appointment | N/A | Not measured | 84.2% (n=160/190) felt comfortable collecting the self-collected sample.  Some of the cited reasons for encountering difficulty with self-collection: being scared they might injure their baby (1.1%, n=2/190), and finding it unpleasant to handle their own genitals (1.1%, n=2/190) | Not measured | Not measured | Not measured | Not measured | Not measured | Not measured | Not measured | 72.6% (n=138/190) reported experiencing no difficulties with self-collection | 27.3% (n=52/190) of participants reported encountering difficulty with self-collection, with some of the cited reasons being: not knowing if they are doing it correctly (16.8% n=32/190), difficulty inserting the swab into both holes (11%, n=21/190), difficulty due to the presence of haemorrhoids (3.1%, n=6/190), not easily locating the holes (1.6%, n=3/190), and difficulty handling the sampling instrument (1.1%, n=2/190). |
| **Price 2006 (19)** | 14.5% (n= 56/386) | 54.8% (n=17/31) preferred sampling to be performed by the healthcare provider; 68.2% (n=15/22) felt uncomfortable with self-collection; 16.1% (n=5/31) reported feeling physically unable to | N/A | Not reported | Not measured | 81.5% (n=251/308) rated the acceptability of self-collection as totally or somewhat acceptable | 22.8% | Not measured | 27.3% | Not measured | Not measured | Not measured | Not measured | Not measured | 14.2% (n=47/330) were concerned about not doing the self-collection correctly |
| **Salvesen 1999 (34)** | Not measured | Not measured | N/A | N/A | A proportion of participants with positive test results and a proportion with negative results were given antibiotics, but these data were not disaggregated by whether these test results were from self-collected or provider-collected samples | Not measured | Not measured | Not measured | Not measured | Not measured | Not measured | Not measured | Not measured | Not measured | Not measured |
| **Seto 2019 (35)** | Not measured | Not measured | N/A | No | 100% | Not measured | 13.0% | Of those who preferred self-sampling (13.0%, n=54/416), 229 reasons were listed, which included less pain (17.5%, n=40/229) and more privacy (43.7%, n=100/229).  Of the participants who didn't prefer provider-collection, 53 reasons were listed, which included fear of pain (26.4%, n=14/53) and feeling of less privacy (54.7%, n=29/53) | 43.3% | Of the participants who didn't prefer self-sampling, 187 reasons were listed, which included fear of pain (17.6%, n=33/187). Of those who preferred provider-collection (43.3%, n=180/416), 355 reasons were listed, which included less pain (8.5%, n=30/355). | Not measured | Not measured | Not measured | Participants rated themselves with a confidence score for self-collection; the mean confidence score was 6.08/10 (± 2.4).  More convenience was listed as a reason for preferring self-collection (56.8%, n=130/229) and as one of the reasons for preferring provider-collection 13.2% (n=47/355), and inconvenience was listed as one of the reasons for not preferring provider-collection (43.4%, n=23/53). | 66.6% (n=273/410) of participants had perceived difficulty with self-collection. Of listed reasons for having difficulty with self-collection these included: difficulty placing the swab into the anus (81.6%, n=226/277) or vagina (15.9% (n=44/277), difficulty identifying the vagina and anus (5.4%, n=15/277), difficulty placing the samples in the container (2.9%, n=8/277), difficulty labelling the samples (0.7%, n=2/277), fear of procedure leading to difficulty (6.9%, n=19/277), and fear of pain leading to difficulty (21.7%, n=60/277). Participants had been provided with written information sheets and a video on self-collection; 88.4% (n=358/405) of participants found the written information sheet useful, and 96.1% (n=393/409) found the video helpful.  Of those who did not prefer self-collection, reasons listed included inconvenience n=40/187 (22.5%), and procedure difficulty n=36/187 (19.3%). |
| **Spieker 1999 (36)** | Not measured | Not measured | N/A | N/A | Not measured | Not measured | Not measured | Not measured | Not measured | Not measured | Not measured | Not measured | Not measured | Not measured | Not measured |
| **Torok 2000 (38)** | Not measured | Not measured | N/A | No | 100% | Not measured | 58.0% | Not measured | Not measured | Not measured | Not measured | Not measured | Not measured | Not measured | Not measured |
